# Cancer-associated fibroblasts in early-stage lung adenocarcinoma correlate with tumor aggressiveness

**DOI:** 10.1038/s41598-023-43296-3

**Published:** 2023-10-17

**Authors:** Georgii Vasiukov, Yong Zou, Maria-Fernanda Senosain, Jamshedur S. M. Rahman, Sanja Antic, Katherine M. Young, Eric L. Grogan, Michael N. Kammer, Fabien Maldonado, Cynthia A. Reinhart-King, Pierre P. Massion

**Affiliations:** 1 Department of Biomedical Engineering, School of Engineering, Vanderbilt University, Nashville, TN USA; 2 Division of Pulmonary and Critical Care Medicine, Vanderbilt University Medical Center, Nashville, TN USA; 3 Division of Thoracic Surgery, Vanderbilt University Medical Center, Nashville, TN USA

**Keywords:** Non-small-cell lung cancer, Cancer genomics, Cancer microenvironment

## Abstract

Lung adenocarcinoma (LUAD) is the predominant type of lung cancer in the U.S. and exhibits a broad variety of behaviors ranging from indolent to aggressive. Identification of the biological determinants of LUAD behavior at early stages can improve existing diagnostic and treatment strategies. Extracellular matrix (ECM) remodeling and cancer-associated fibroblasts play a crucial role in the regulation of cancer aggressiveness and there is a growing need to investigate their role in the determination of LUAD behavior at early stages. We analyzed tissue samples isolated from patients with LUAD at early stages and used imaging-based biomarkers to predict LUAD behavior. Single-cell RNA sequencing and histological assessment showed that aggressive LUADs are characterized by a decreased number of ADH1B^+^ CAFs in comparison to indolent tumors. ADH1B^+^ CAF enrichment is associated with distinct ECM and immune cell signatures in early-stage LUADs. Also, we found a positive correlation between the gene expression of ADH1B^+^ CAF markers in early-stage LUADs and better survival. We performed TCGA dataset analysis to validate our findings. Identified associations can be used for the development of the predictive model of LUAD aggressiveness and novel therapeutic approaches.

## Introduction

Lung cancer is the leading cause of cancer death in the United States and worldwide, killing 1.8 million individuals every year^1^. The high death rate of lung cancer is mostly associated with its late diagnosis, and low-dose chest CT screening helps to detect lesions early and significantly reduces lung cancer mortality^2,3^. Lung adenocarcinoma (LUAD) is of particular interest because it is the predominant histological type of lung cancer within the U.S. and is rapidly growing in other countries, and it exhibits a wide spectrum of behavior that can be indolent or very aggressive characterized by rapid tumor growth, invasion of healthy tissue and metastasis^1,4^. Thus, patients with indolent LUAD at early stages often undergo overdiagnosis and overtreatment, whereas individuals with aggressive tumors require urgent therapeutic procedures. Selection and utilization of the correct treatment strategy requires novel methods for the prediction of LUAD behavior and prognosis. For this purpose, it is necessary to identify and better understand the biological determinants of LUAD aggressiveness.

Biological mechanisms associated with LUAD behavior are still poorly characterized. Several reports have demonstrated that LUAD behavior is associated with the histological subtype of the tumor, anti-cancer immune response, mutational burden, age, and smoking status of the patients^4–6^. Nevertheless, existing predictive models are based on the biology of the tumor cells and ignore other components of the tumor microenvironment which can be specific to LUAD. Cancer behavior is associated with extracellular matrix deposition and remodeling, and cancer-associated fibroblasts (CAFs) play a critical role in these processes^7,8^.

CAF populations in the tumor microenvironment are diverse and characterized by different origins, spatial distribution, and markers^7^. Beyond the functions associated with the ECM, CAFs are involved in a number of different processes: modulation of immune response, tumor cell collective migration and invasion, angiogenesis, and metabolic reprogramming^7,9–14^. For instance, CAFs can produce cytokines that can modulate anti-cancer immune responses^7,15^. However, it is still not clear if CAFs act only as a pro-tumorigenic factor or, depending on the context, they can play an anti-tumorigenic role^12–14^. Context-dependent involvement of CAFs in determination of tumor progression can be used for tumor behavior prediction. Recent progress in single-cell technologies gives an opportunity to perform a more detailed and precise analysis of CAFs in the tumor microenvironment.

Here, we used an orthogonal, previously validated imaging-based biomarkers^16^ to perform stratification of early-stage LUAD patients based on predicted indolent or aggressive behavior of the tumors. We performed the analysis of the tumor samples using single-cell RNA sequence (scRNAseq) and histological assessment. We parsed apart separate subpopulations of CAFs in the tumor microenvironment and identified the association between the ADH1B^+^ CAF enrichment of LUAD at early stages and predicted tumor behavior. Our findings were confirmed through the analysis of the TCGA dataset from LUAD patients.

## Materials and methods

### Human specimens

The experimental protocols were approved by the Vanderbilt University Medical Center Institutional Review Board (IRB protocol #000616). All patients involved in the research provided informed consent. Lung adenocarcinoma tissue was isolated from 16 patients during tumor resection at the Vanderbilt University Medical Center (Supplementary Table T1). The inclusion criterion for the patients was the availability of a chest CT scan prior to resection and early stage (stage I and II) lung adenocarcinoma. The cohort was composed of 12 females and 4 males. The median patient age was 71.5 (59–86). All methods were carried out in accordance with relevant guidelines and regulations.

### Tumor stratification

**Figure 1:**
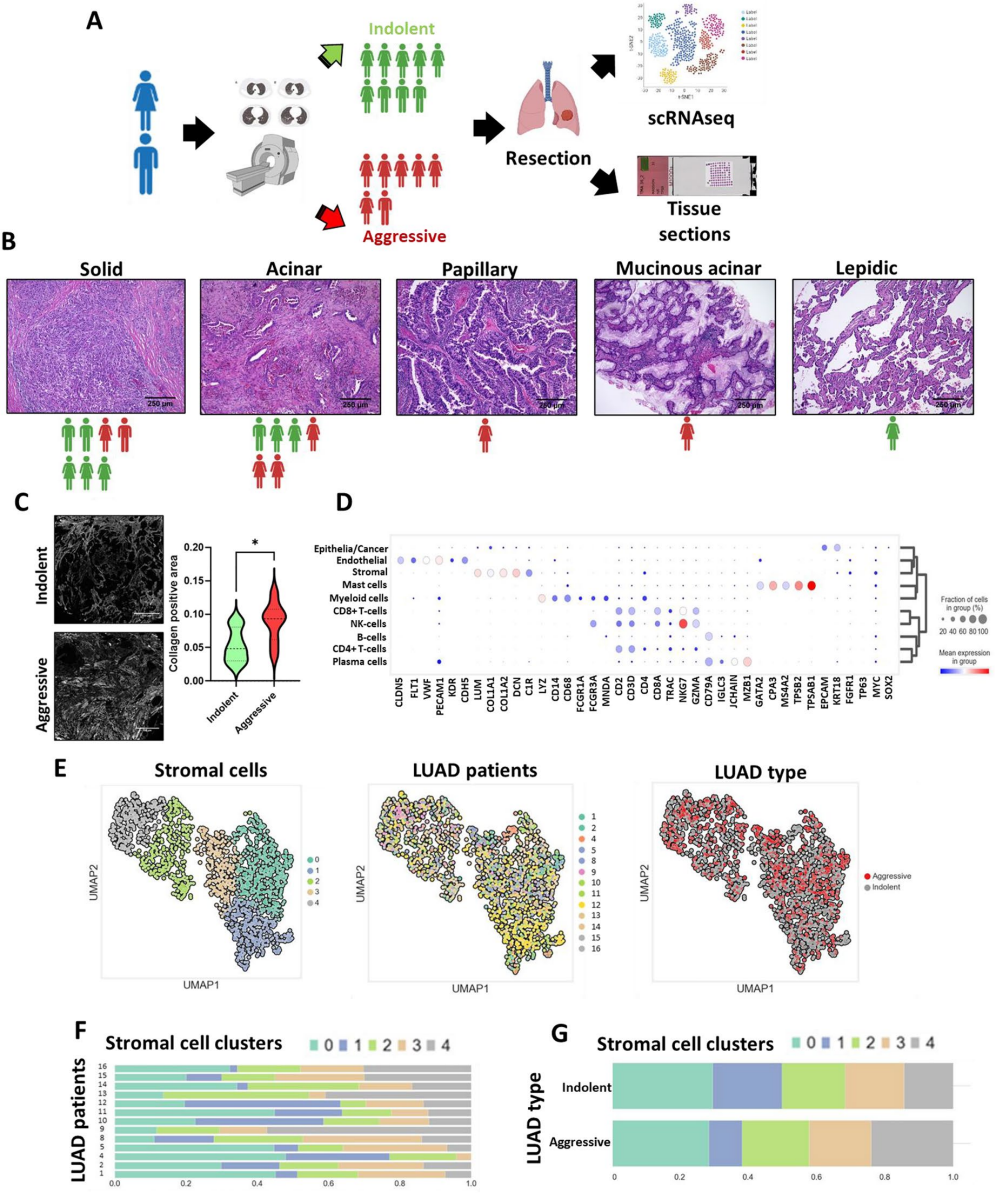
Histological subtype and cellular composition of indolent and aggressive LUADs. (**A**) Design of the experiment. Using chest CT-scan analysis, LUAD patients were stratified into 2 groups with predicted indolent and aggressive tumor behavior. (Illustrations are created with BioRender.com.) (**B**) Representative images of histological subtypes identified in the experimental cohort (H&E staining, indolent n = 8, aggressive n = 7). The icons demonstrate distribution of the patients from indolent (green) and aggressive (red) groups with respect to identified histological subtypes of the LUAD tumor tissue. (**C**) Representative images of CHP staining showing deposition of collagen in the tumor tissue isolated from patients with indolent and aggressive LUADs. Corresponding quantification of collagen positive area in the LUAD tissue isolated from the patients (violine plot shows median collagen positive area; indolent n = 9, aggressive n = 7; *—*p* < 0.05). (**D**) Expression of canonical marker genes associated with major cell populations in the tumor microenvironment across identified cell clusters (scRNAseq data). (**E**) UMAP representation of the stromal cell population (indolent n = 6, aggressive n = 7). Projection of identified clusters within stromal cell populations (left). Projection of samples isolated from LUAD patients within stromal cell populations (middle). Projection of samples isolated from indolent and aggressive LUADs within stromal cell subpopulation (right). (**F**) Proportion of stromal cell subpopulations in relation to patients. (**G**) Proportion of stromal cell subpopulations in indolent and aggressive LUADs.

Tumors were classified as “indolent” and “aggressive” using Computer-Aided Nodule Assessment and Risk Yield (CANARY) software as described previously^17,18^. The stratification strategy was based on the calculation of the Score Indicative of Lung Cancer Aggression (SILA). SILA ranges from 0 to 1 and is determined by 9 radiologically distinct exemplars^16^. Using a variety of geometrical and textural features of lung tumors, the CANARY is able to distinguish in situ and invasive carcinomas, predict the invasiveness of the tumor, as well as LUAD patient survival and mortality at the early stage of progression. We set a SILA threshold at 0.5 to distinguish patients with promising and poor predicted prognoses. The group with a SILA score lower than 0.5 was assigned as indolent, and the patients with a SILA score higher than 0.5 were assigned as aggressive. The indolent group was composed of 9 patients and the aggressive group was composed of 7 patients (Fig. 1A).

### Tumor tissue isolation and processing

The tumor tissue samples were placed in cold PBS immediately after the surgery and divided into two parts for histological analysis and scRNAseq (see scRNAseq and histological samples preparation paragraphs below). Tissue samples for histological assessment have been examined by pathologist and placed in a 10% neutral buffered formalin for 24 h at room temperature for fixation. The samples for scRNAseq have been dissociated into a single-cell suspension within one hour. To prepare the digestive media, 1 mg/ml Collagenase I (Sigma Aldrich, USA) and 1 mg/ml of DNAse I (Sigma Aldrich, USA) were mixed in DMEM media (Thermo Fisher Scientific, USA). Minced tumor tissue was incubated in the media in a shaking water bath for 1 h at 37 °C. After incubation, tumor tissue was disrupted using a syringe pestle and filtered through 70 um strainers to remove debris. The suspension was additionally filtered using a 30 um strainers. Single-cell suspensions were cryopreserved for long-term storage. Cells were counted, and their viability was assessed before and after cryopreservation.

### Single-cell RNA sequencing (scRNAseq) sample preparation and data acquisition

Cryopreserved single-cell suspensions prepared from isolated LUAD tissues were used for scRNAseq. Dead cells were removed using a MACS Dead Cell Removal Kit (Miltenyi Biotec, Germany). From each sample, 5000–10,000 cells were used for scRNAseq using a 10× Genomics platform (10× Genomics, USA). Library preparation was performed in accordance with the manufacturer’s protocol. The libraries were sequenced using a NovaSeq 6000 with 150 bp paired reads. Feature matrices preparation was performed using 10× Genomics Cell Ranger software (10× Genomics, USA). The library was aligned to the GRCh38 reference genome. The analysis of the scRNAseq dataset was completed using a Python 3 package, ScanPy^19^. Cells with either less than 250 or more than 4000 genes detected were filtered out. Additionally, cells with a percentage of mitochondrial genes higher than 17% were excluded from the analysis. Cell populations were annotated in accordance with gene expression patterns that have been demonstrated in prior literature. For dimensionality reduction, we used the Uniform Manifold Approximation and Projection algorithm (UMAP)^20^. The Leiden algorithm was used for the clustering of cell populations^21^. In the samples isolated from patients 3, 6, and 7, no stromal cells were identified. The composition of patient samples' cells could have been affected by procedures related to sample isolation, tumor tissue dissociation, or cryopreservation.

### Patient tumor tissue preparation and histologic staining

After fixation tissue samples have been embedded in paraffin. Next, 5–7 um tissue sections were prepared. The tissue samples were examined by a pathologist to determine the histological subtypes of lung adenocarcinomas. Hematoxylin and eosin (H&E) and multiplex immunofluorescent (MxIF) staining were used for tissue analysis. MxIF staining was used to identify tumor cells and ADH1B^+^ CAFs. Epithelial/tumor cells were detected using anti-PanCK-Alexa Fluor 488 antibodies (eBioscience, USA), and ADH1B^+^ CAFs were visualized using rabbit anti-human antibodies (Jackson ImmunoResearch, USA). Nuclei were located using Gold Antifade Mountant with DAPI (Thermo Fisher Scientific, USA). For visualization of collagen in the tissue samples we used CHP–Collagen Hybridizing Peptide (3Helix, United States) staining labeled with Cy3 dye. Tissue images were obtained using the Keyence BZ-X710 microscope (KEYENCE, USA) using a 10× objective with a Texas Red filter (excitation wavelength 560/55 nm, emission wavelength 645/75 nm), a GFP filter (excitation wavelength 480/30 nm, emission wavelength 535/40 nm), and a DAPI filter (excitation wavelength 350/50 nm, emission wavelength 460/50 nm). Tissue image analysis was performed using ImageJ^22^ and QuPath^23^ software. LUAD histological subtypes were defined by a pathologist. When multiple subtypes were identified in one tissue one subtype was assigned as predominant based on the area of tissue occupied. To estimate collagen deposition and assess cell numbers, we analyzed at least 10 areas for each sample.

### TCGA dataset acquisition and analysis

We used R programming language and appropriate packages (“TCGAbiolinks”^24^, “limma”^25^, and “edgeR”^26–28^) to download and analyze a TCGA dataset. The data of 419 patients with LUADs at stages I and II (191 males and 228 females) were assessed. The cohort was composed of 323 Caucasians, 6 Asians, and 43 African Americans, and 47 samples with missing racial and ethnic information. All other parameters such as sex, age, treatment, histological type, and recurrence, were not taken into account. Specifically for Fig. 4C we assessed TCGA LUAD samples at stage I (n = 294), stage II (n = 125), stage III (n = 84), stage IV (n = 26). All other inclusion criteria were not applied. For the analysis, we have used transcriptome profiling data obtained using bulk RNA sequencing.

### Survival analysis

Survival analysis of the downloaded TCGA dataset has been performed using “survival” package^29^. The package contains the core survival analysis routines, including Kaplan–Meier and Aalen–Johansen (multi-state) curves, and Cox models. In addition, stratified Kaplan–Meier plots were generated using available web Kaplan–Meier plotter^30^. The tool uses information downloaded from The Cancer Genome Atlas (TCGA), Gene Expression Omnibus (GEO), and European Genome-Phenome Archive (EGA). LUAD samples at stage I were analyzed using this tool. The dataset was not adjusted to specific criteria such as sex, age, treatment, recurrence etc.

### Cell enrichment analysis

To calculate cell enrichment score for bulk RNAseq data (TCGA dataset) we utilized the CIBERSORT algorithm (https://cibersortx.stanford.edu). The algorithm implements linear support vector regression to calculate the relative levels of cells. Signature matrix for 3 CAF subpopulations were generated using our scRNAseq dataset. For estimation of immune cell enrichment score we used predefined signature matrix.

### Statistical analysis

Statistical analyses and plot generation were performed using GraphPad Prism 9 (https://www.graphpad.com), Python 3 (https://www.python.org) and R 4.2.1 (https://www.r-project.org). One-way ANOVA followed by Dunnett’s procedure was used for multiple comparisons between groups. A two-sided comparison was performed using a two-sample t test or a Wilcoxon Rank-Sum test as appropriate. For correlation analysis, we used Pearson correlation coefficient. The Kaplan–Meier method was used to visualize survival plots and a log rank test was used for an estimation of statistical significance. All tests were considered statistically significant at a two-sided 5% level.

## Results

### Tissue structure and cellular composition of indolent and aggressive LUADs at early stages

Histological assessment of the tumors in the cohort identified 7 solid, 6 acinar, 1 papillary, 1 mucinous acinar, and 1 lepidic histological subtypes (Fig. 1B, Supplementary Table T1). Part of the samples (6 of 16) had a mixed structure with 1 predominant structural pattern. In our cohort, tumor samples with predicted aggressive behavior had solid (2 of 7), acinar (3 of 7), papillary (1 of 7), and mucinous acinar (1 of 7) patterns (Fig. 1B). Indolent LUADs had solid (5 of 9), acinar (3 of 9) and lepidic (1 of 9) predominant histological patterns (Fig. 1B, Supplementary Table T1). A broad variety of histological subtypes in the LUADs complicates the utilization of histological patterns alone for the prediction of LUAD behavior, especially at early stages. However, we observed increased collagen deposition in LUADs with predicted aggressive behavior (Fig. 1C). It is well known that CAFs play a crucial role in the processes of ECM deposition and remodeling during tumor development. We decided to investigate the CAF subpopulations in the tumor microenvironment of indolent and aggressive LUADs using scRNAseq.

A droplet-based scRNAseq method (10× Genomics) was used to perform a comprehensive analysis of the CAF population in the tumor microenvironment. This analysis identified 43,207 cells with distinct transcriptomes. 21,140 cells (48.93%) were obtained from indolent LUADs and 22,067 (51.07%) were from aggressive LUADs. Using graph-based uniform manifold approximation and projection algorithm (UMAP)^20^ and Leiden clustering algorithm^21^, 26 cell clusters were identified. A set of canonical cell markers were used to assign all detected cell clusters to 10 cell populations. The following cell populations were identified: epithelial/cancer cells (EPCAM^+^, CK18^+^), endothelial cells (PECAM1^+^, VWF^+^), stromal cells (LUM^+^, DCN^+^), mast cells (CPA3^+^, TPSAB1^+^), myeloid cells (LYZ^+^, CD68^+^), CD8^+^ T cells (CD3D^+^, CD8A^+^), NK cells (FCGRA1^+^, NKG7^+^), B-cells (CD79A^+^), CD4^+^ T-cells (CD3D^+^, CD4^+^), and plasma cells (CD79A^+^, MZB1^+^) (Fig. 1D). The stromal population was composed of 986 cells from indolent LUADs (57.2%) and 673 from aggressive tumors (42.8%). To identify the CAF population, an additional clustering of the stromal cell population was performed, which identified 5 additional subclusters of stromal cells (Fig. 1E). The comparison of the proportions of stromal cell clusters demonstrated high heterogeneity between patient samples as well as within the indolent and aggressive groups. (Fig. 1F,G).

### CAF population in indolent and aggressive LUADs is comprised of 3 distinct subpopulations

**Figure 2:**
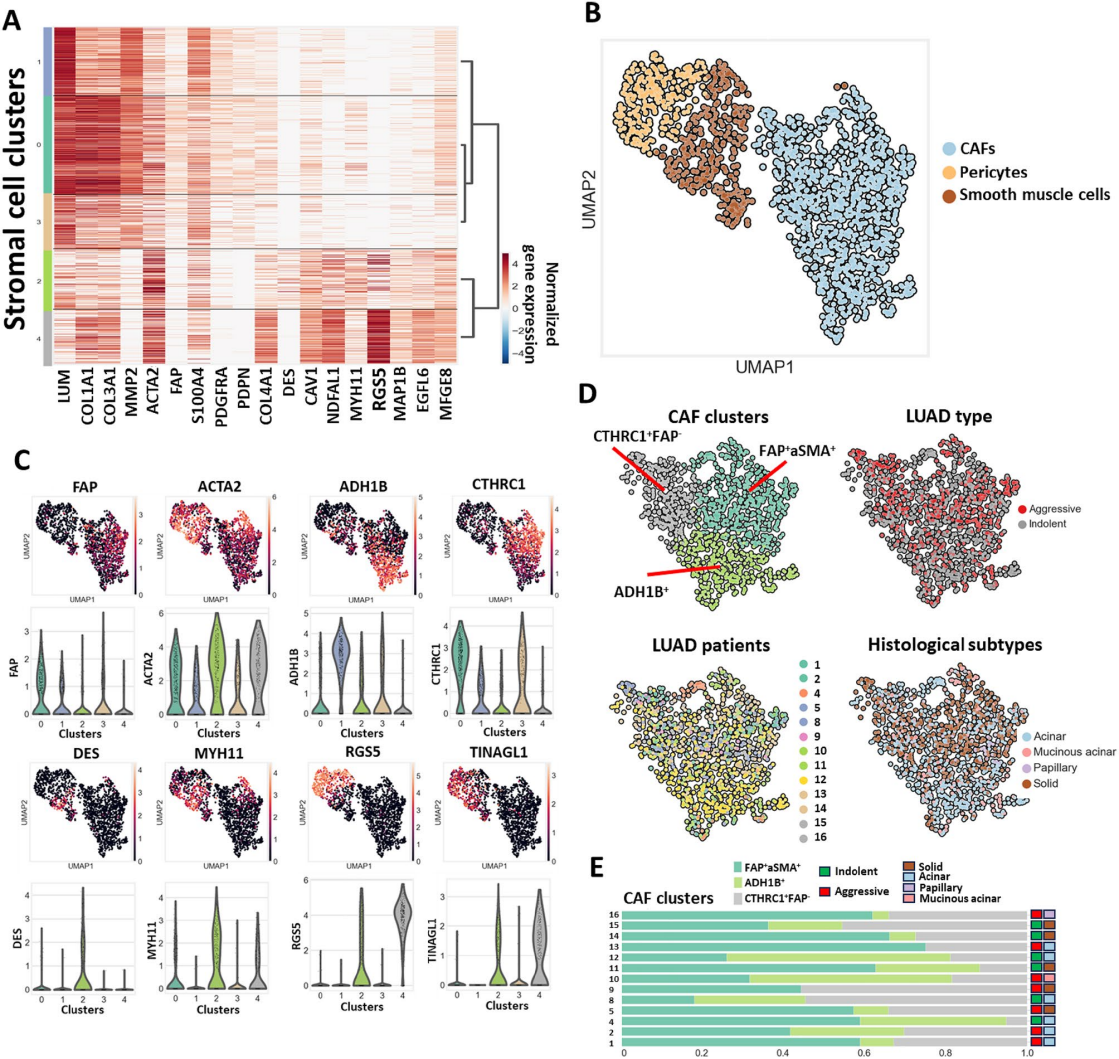
CAF population in indolent and aggressive LUADs. (**A**) Expression of canonical genes associated with CAFs, smooth muscle cells, and pericytes. Dendrograms are generated in accordance with hierarchical clustering. Heatmap was conducted using Python 3 (https://www.python.org). (**B**) UMAP representation of stromal cell subpopulations. Subpopulations of CAFs, pericytes, and smooth muscle cells are highlighted by color. (**C**) Expression of canonical marker genes of CAFs (FAP^+^ACTA2^+^; ADH1B^+^, CTHRC1^+^FAP^-^), pericytes (RGS5^+^TINAGL^+^), and smooth muscle cells (DES^+^MYH11^+^) in stromal cell subpopulations. UMAP representations show expression of canonical marker genes across identified stromal cell clusters. Violin plots show expression of genes across identified stromal clusters. (**D**) UMAP representation of CAF populations. Identified subclusters in CAF population (top left). Distribution of cells from patients with indolent and aggressive LUADs within CAF population (top right). Distribution of cells from different patient samples within stromal cell subpopulation (bottom left). Distribution of cells from different histological subtypes within CAF population (bottom right). (**E**) Proportion of CAF subpopulations in relation to patients with indolent and aggressive LUADs (scRNAseq data).

To classify the subpopulations of stromal cells and distinguish clusters of CAFs, the expression of LUM, COL1A1, COL3A1, MMP2, ACTA2, FAP, S100A4, PDGFRA, PDPN, COL4A1, DES, CAV1, NDUFA4L2, MYH11, RGS5, MAP1B, EGFL6, and MFGE8 was analyzed. When coupled with hierarchical clustering, the data indicated that clusters 2 and 4 have gene expression patterns associated with pericytes and smooth muscle cells (Fig. 2A). Cluster 4 is characterized by high expression of RGS5 and TINAGL1, which are well-known markers of pericytes. Cluster 2 demonstrated expression of DES and MYH11 and was recognized as smooth muscle cells (Fig. 2B,C). We noticed that a small number of cells from stromal clusters 2 and 4 express several CAF markers (such as LUM and COL1A1). This can be explained by cellular plasticity of stromal cells, functional convergence, and similar microenvironmental influences. Clusters 0, 1, and 3 were determined to be CAFs. Further exploration showed that 663 CAFs (62.8%) were obtained from indolent group and 393 CAFs (37.2%) were obtained from aggressive group. Cluster 0 was assigned as FAP^+^ACTA2^+^ CAFs. Cluster 1 was identified as ADH1B^+^ CAFs. Since normal fibroblasts also express ADH1B, we employed another marker of normal fibroblasts, CD10 (MME), to corroborate that the identified ADH1B^+^ population consists of CAFs. Our findings revealed that the ADH1B^+^ cluster lacks or demonstrates very low CD10 expression, further confirming that the cells within this cluster are indeed CAFs and not normal fibroblasts (Supplementary Fig. S1). This approach has also been utilized by Grout et al.^31^. Cluster 3 was assigned as CTHRC1^+^FAP^-^ CAFs (Fig. 2D). We also noticed heterogeneity in CAF cluster composition among patients in both the indolent and aggressive groups. This variability might indicate different levels of biological processes related to CAF functions in the tumor microenvironment, such as the deposition and remodeling of ECM, modulation of immune processes, and angiogenesis (Fig. 2D,E).

### CAF subpopulations are characterized by different function and role in the tumor progression and aggressiveness

**Figure 3:**
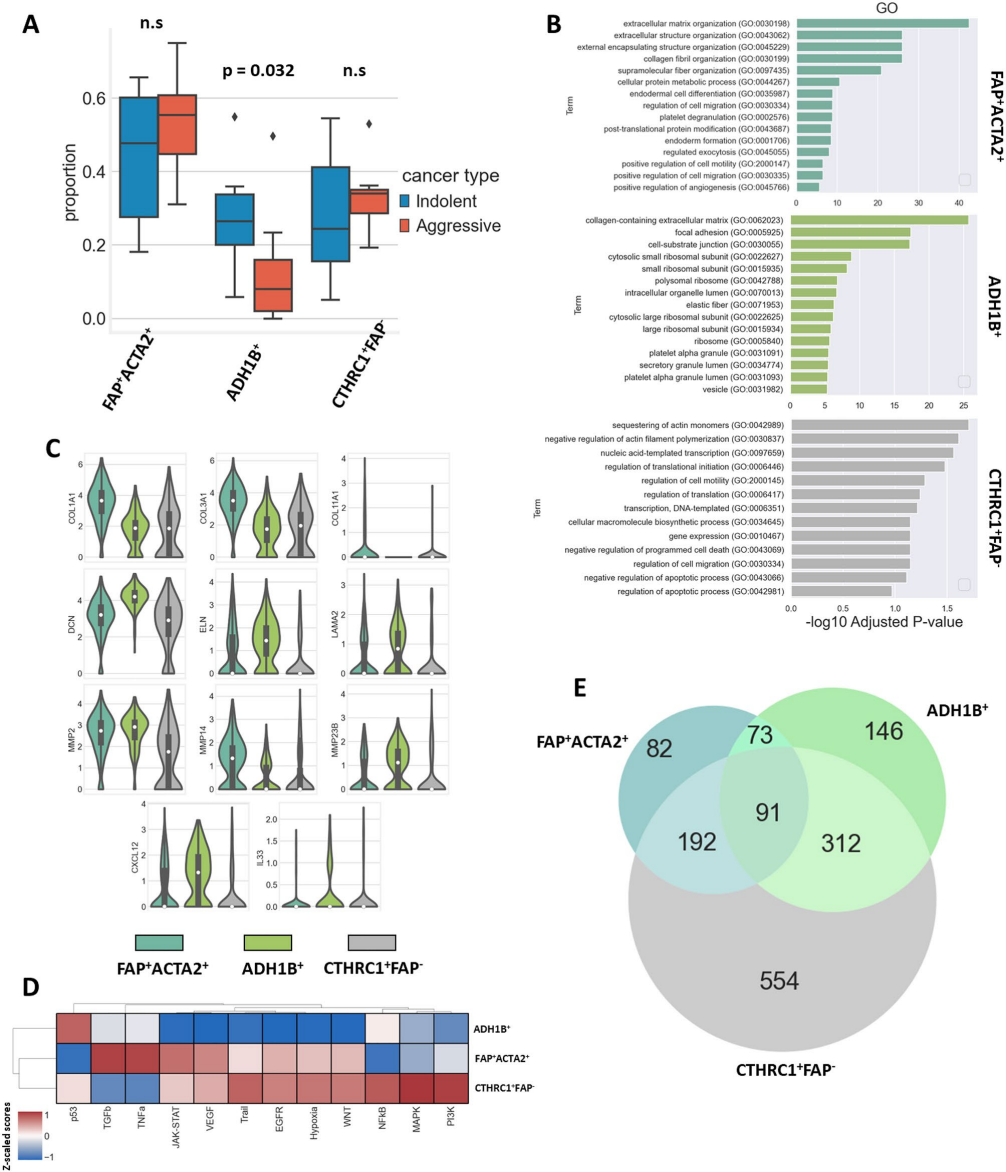
CAF subpopulation proportions and functions. (**A**) Proportion of CAF subpopulations in indolent and aggressive LUADs (indolent n = 6, aggressive n = 7, *p* = 0.032, outliers are highlighted by diamonds). (**B**) GO enrichment analysis of identified CAF subpopulations. (**C**) Violin plots demonstrate expression of genes associated with functions of identified CAF subpopulations. (**D**) Heatmap demonstrates activity of canonical signaling pathways across identified CAF subpopulations. Dendrograms are generated in accordance with hierarchical clustering. Heatmap was conducted using Python 3 (https://www.python.org). (**E**) Venn diagram shows intersection of differentially expressed genes in identified CAF subpopulations.

The subpopulation of FAP^+^ACTA2^+^ CAFs was composed of 276 cells (59.6%) from indolent LUADs and 187 cells (40.4%) from aggressive carcinomas. The cluster of ADH1B^+^ cells was formed by 234 (71.8%) and 92 (28.2%) CAFs from the indolent and aggressive tumors, respectively. The group of CTHRC1^+^FAP^−^ CAFs consists of 153 cells (56.2%) from indolent and 119 cells (43.8%) from aggressive LUADs. The comparison of the average proportions between different types of CAFs revealed that aggressive LUADs are characterized by a decreased proportion of cells that form the ADH1B^+^ CAF cluster (*p* = 0.032) (Fig. 3A).

Gene ontology (GO) enrichment analysis indicates that CAFs from the FAP^+^ACTA2^+^ cluster were mostly involved in processes associated with the deposition of ECM proteins and matrix remodeling (Fig. 3B). Indeed, that subpopulation of CAFs demonstrated increased expression of COL1A1, COL3A1, COL11A1, and FN1. Also, this cluster was characterized by high expression of MMP2 and MMP14 (Fig. 3C). In addition, the analysis of genes associated with different signaling pathways showed that the FAP^+^ACTA2^+^ cluster was characterized by increased activity of TGFβ pathway (Fig. 3D).

GO enrichment analysis indicates that the ADH1B^+^ subpopulation also has been involved in the deposition and remodeling of ECM structures (Fig. 3B). However, in comparison to the FAP^+^ACTA2^+^ cluster, it had lower expression of COL1A1, COL3A1, and MMP14. On the other hand, we identified increased expression of ELN, LAMA2, and DCN in ADH1B^+^ CAFs. Further analysis revealed that the ADH1B^+^ cluster was also characterized by enhanced expression of cytokines such as CXCL12 and IL33 (Fig. 3C). The analysis of genes associated with signaling pathways showed that ADH1B^+^ cluster was characterized by decreased activity of JAK-STAT, VEGF, Trail, EGFR, hypoxia, and WNT pathways in comparison to other CAF clusters (Fig. 3D).

Increased activity of the JAK-STAT, VEGF, Trail, EGFR, hypoxia, and WNT signaling pathways was observed in both the CTHRC1^+^FAP^-^ and FAP^+^ACTA2^+^ CAF subpopulations. On the other hand, the CTHRC1^+^FAP^-^ subpopulation had decreased activity of the TGFβ and TNFa pathways, resembling the activity of the ADH1B^+^ CAFs (Fig. 3D). The Venn diagram shows that CTHRC1^+^FAP^-^ CAFs share 283 and 403 similar differentially expressed genes with FAP^+^ACTA2^+^ and ADH1B^+^ CAFs respectively, whereas FAP^+^ACTA2^+^ and ADH1B^+^ subpopulations only share 164 overlapping genes (Fig. 3E). The expression of COL1A1 and COL3A1 in the ADH1B^+^ cluster was close to that of the CTHRC1^+^FAP^-^ cluster. The expression of DCN, ELN, and LAMA2 in FAP^+^ACTA2^+^ CAFs was similar to that of CTHRC1^+^FAP^-^ CAFs. The expression of CXCL12 and IL33 cytokines in the CTHRC1^+^FAP^-^ subpopulation was decreased in comparison to that of both the ADH1B^+^ and FAP^+^ACTA2^+^ clusters (Fig. 3C). Genes associated with iCAF, myCAF, and apCAF profiles revealed that all 3 identified CAF subpopulations exhibit similarity to myCAF cells (Supplementary Fig. S2). Altogether, the analysis of the gene expression profile, the activity of signaling pathways, and the Venn diagram of overlapping differentially expressed genes across subtypes may indicate that the three observed subpopulations of CAFs could be transient forms of each other. Nevertheless, this question requires further investigation.

### ADH1B^+^ CAF markers are prognostic for patients with lung adenocarcinoma at early stages

**Figure 4:**
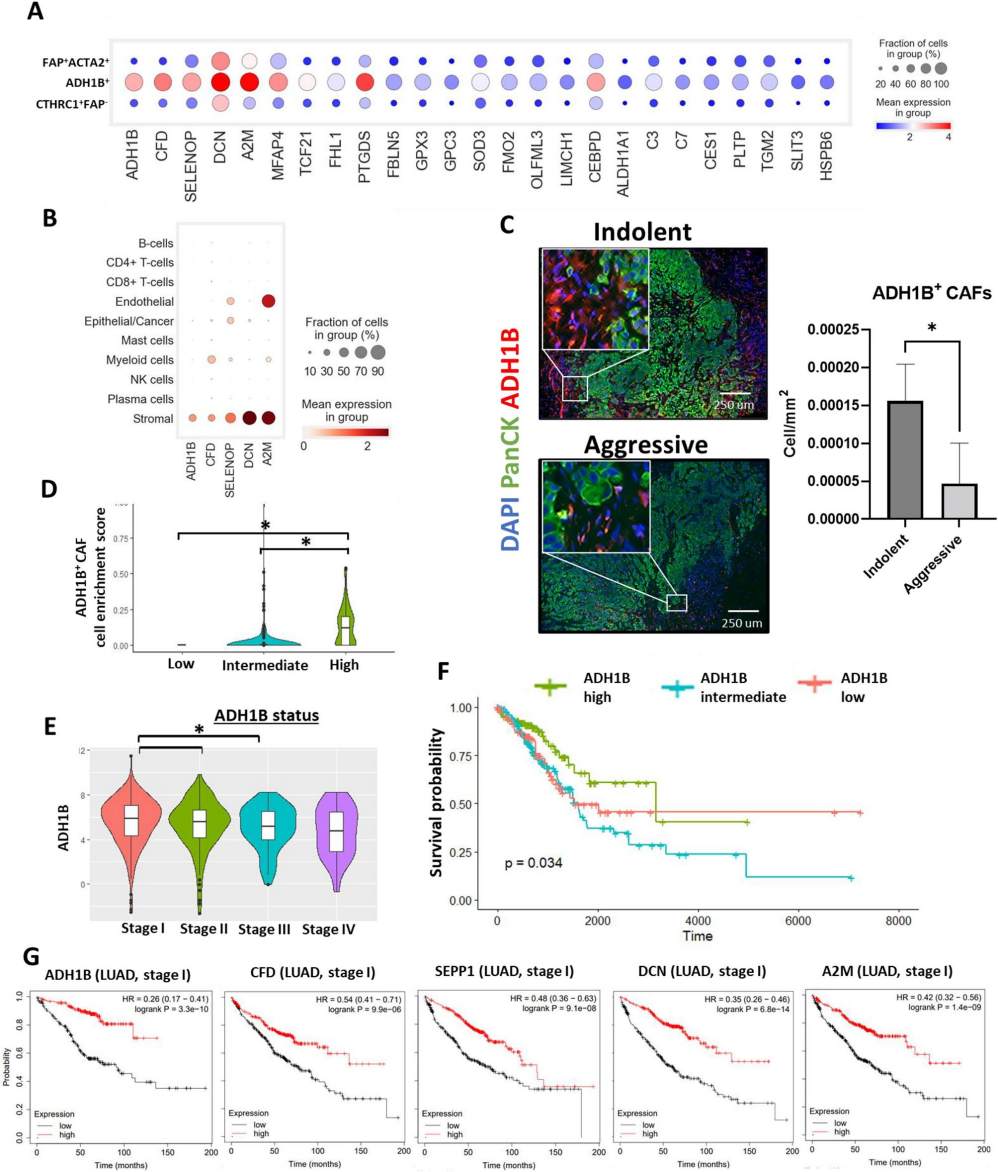
ADH1B^+^ CAF markers are prognostic for patients with lung adenocarcinoma at early stages. (**A**) Expression analysis of the top 25 differentially expressed genes related to the ADH1B+ CAF cluster across all CAF clusters. (**B**) Gene expression of ADH1B^+^ CAF markers across all identified subpopulations of cells. (**C**) Representative images of MxIF staining showing expression of PanCK and ADH1B in LUAD tissue isolated from patients with predicted indolent and aggressive tumor behavior. Corresponding cellular density of ADH1B^+^ CAFs (cell/mm^2^) in the LUAD tissue extracted from the patients with predicted indolent or aggressive tumor behavior (indolent n = 7, aggressive n = 6, *—*p* < 0.05, data showed as mean ± SEM). (**D**) Cell enrichment analysis (CIBERSORT) of LUAD patient samples (stage I and II, TCGA dataset). Violin plots demonstrate ADH1B^+^ CAF enrichment scores in TCGA samples with low (n = 105), intermediate (n = 209), and high (n = 105) expression of ADH1B (*p < 0.05). (**E**) Gene expression level of ADH1B in LUAD samples (TCGA dataset) at different stages of development (independent samples, stage I n = 294, stage II n = 125, stage III n = 84, stage IV n = 26, *p < 0.05). (**F**) Kaplan–Meier analysis of the overall survival of LUAD patients with low (n = 105), intermediate (n = 209), and high (n = 105) expression of ADH1B (stage I and II, TCGA dataset). (**G**) Kaplan–Meier analysis (KM-plotter data) of the overall survival of patients with LUADs at stage I and characterized by low and high expression of top marker genes associated with ADH1B^+^ CAF subpopulation (ADH1B (low n = 185, high n = 185), CFD (low n = 288, high n = 289), SEPP1 ((low n = 288, high n = 289)), DCN (low n = 288, high n = 289), and A2M (low n = 288, high n = 289).

As the proportion of ADH1B^+^ subpopulation in the indolent group was significantly increased, we decided to focus our attention on this subtype of CAFs. A ranking of the differentially expressed genes was performed and, in addition to ADH1B, this ranking identified CFD, DCN, SEPP1 (SELENOP), A2M and MFAP4 as top marker genes of this subpopulation of cells (Fig. 4A). In addition, the expression of these genes among other populations of cells and other CAF subpopulations was assessed. This expression analysis showed that myeloid cells are characterized by relatively high expression of CFD. Moreover, high expression of SEPP1 (SELENOP) was also observed in endothelial and epithelial/cancer populations. All CAF subpopulations expressed ADH1B, CFD, DCN, SEPP1 (SELENOP), A2M, and MFAP4, but ADH1B^+^ CAFs demonstrated dramatically higher expression of these genes in comparison to other CAFs subtypes (Fig. 4A,B, Supplementary Fig. S3A).

To compare the number of ADH1B^+^ CAFs in indolent and aggressive LUADs MxIF staining was performed. The results obtained by scRNAseq suggested that ADH1B by itself can act as a marker for the identification of this subpopulation of CAFs as its expression is very low in other populations of cells. As we expected, the number of ADH1B^+^ fibroblasts in the tumor tissue of indolent LUADs was significantly higher (FC = 4.02, *p* < 0.05) in comparison to the aggressive group, supporting the results obtained using scRNAseq (Fig. 4C). Next, we have used bulk RNAseq dataset obtained from TCGA and performed cell enrichment analysis using the CIBERSORT algorithm to estimate cell enrichment score of ADH1B CAFs. We found that the expression of ADH1B positively correlates with ADH1B^+^ CAF enrichment score (Fig. 4D). Furthermore, we observed a positive correlation between the expression of ADH1B and other identified ADH1B^+^ CAF markers, including CFD (DF), A2M, MFAP4, SEPP1 (SELENOP), and DCN (Supplementary Fig. S3B).

ADH1B expression is very specific for fibroblast-like cells in the LUAD tumor microenvironment that makes it one of the most important markers for the detection of ADH1B^+^ CAFs (Fig. 4B; Supplementary Fig. S3A). Gene expression of ADH1B in LUADs is associated with tumor development and decreases as tumor progresses (Fig. 4E). We also identified a decrease in the number of ADH1B + CAFs in stage II LUADs compared to stage I LUADs (Supplementary Fig. S3C). Survival analysis demonstrated that high expression of ADH1B is associated with better survival of LUAD patients (stage I and II, *p* = 0.034) (Fig. 4F).

Further analysis identified that other top marker genes of ADH1B^+^ CAFs are associated with better survival of LUAD patients at early stage. We utilized KM-plotter tool (https://kmplot.com) for this purpose. Patients were stratified into two groups based on the expression of several marker genes (high and low): ADH1B, CFD (DF), A2M, SEPP1 (SELENOP), and DCN. The median expression was used as separation point. Patients with high expression of ADH1B (logrank *p* < 0.01, HR = 0.26), CFD (DF) (logrank *p* < 0.01, HR = 0.54), SEPP1 (SELENOP) (logrank *p* < 0.01, HR = 0.48), DCN (logrank *p* < 0.01, HR = 0.35), and A2M (logrank *p* < 0.01, HR = 0.42) demonstrated better survival at stage I of LUAD (Fig. 4G). Altogether these results indicate that the presence of ADH1B^+^ CAFs in the LUAD tissue is associated with the indolent behavior of the tumor and better overall survival of patients.

### Biological processes, immune cell signature, and ECM deposition processes associated with ADH1B^+^ CAF tissue enrichment

**Figure 5:**
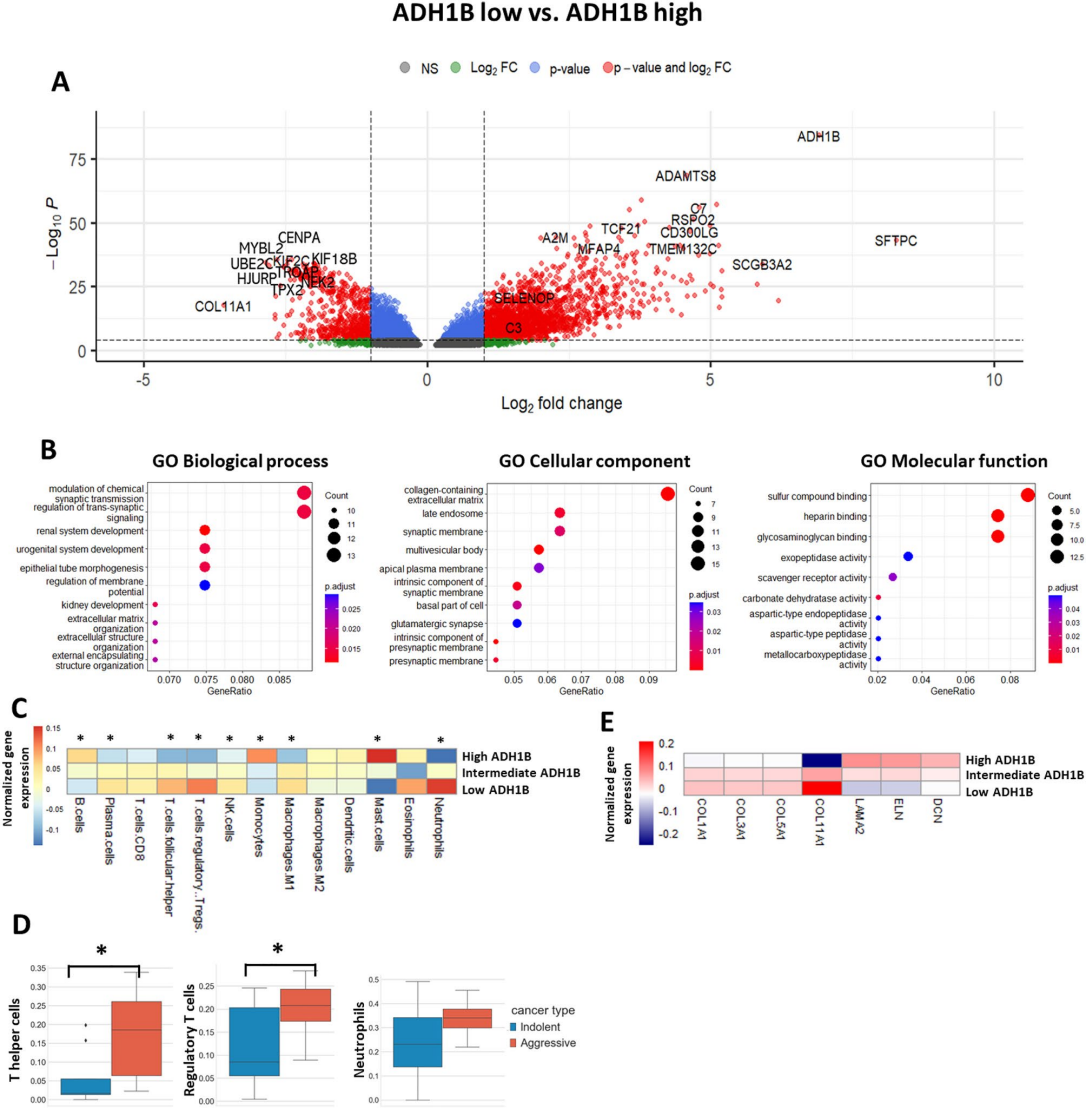
Biological processes, immune cell signature, and ECM deposition processes associated with presence of ADH1B^+^ CAF in the LUAD tissue. (**A**) Volcano plot shows the differentially expressed genes among groups of LUAD patients (stages I and II, TCGA dataset) with low (n = 105) and high (n = 105) expression of ADH1B. (**B**) Gene ontology enrichment analysis of differentially expressed genes in relation to biological processes, cellular component, and molecular function (TCGA dataset). (**C**) Heatmap demonstrates immune cell enrichment scores (CIBERSORT) in the samples with low (n = 105), intermediate (n = 209), and high (n = 105) expression of ADH1B (*—*p* < 0.05, low vs. high, TCGA dataset). Heatmap was conducted using R 4.2.1 (https://www.r-project.org). (**D**) Proportions of regulatory T cells, T helper cells, and neutrophils (scRNAseq data) in LUAD patient samples with predicted indolent (n = 6) and aggressive (n = 7) tumor behavior (*—*p* < 0.05). (**E**) Expression of ECM proteins in the samples with low (n = 105), intermediate (n = 209), and high (n = 105) expression of ADH1B (TCGA dataset). Heatmap was conducted using R 4.2.1 (https://www.r-project.org).

Since we identified that ADH1B expression positively correlates with ADH1B^+^ CAF enrichment (Fig. 4D), and it is very specific marker for ADH1B^+^ CAFs in the tumor microenvironment (Fig. 4A, Supplementary Fig. S3A), we decided to stratify TCGA samples into ADH1B low, intermediate and high groups. To investigate gene expression profile of LUAD samples with low and high expression of ADH1B, we performed differential gene expression analysis. We found ADH1B^+^ CAF markers such as A2M and MFAP4 among the top upregulated genes in ADH1B high group. Moreover, this group of LUAD samples demonstrated the enhanced expression of genes associated with better prognosis such as SFTPC, SCGB3A2, GRIA1, CYP4B1, RSPO2, CD300LG, ADAMTS8, and TMEM132C (Fig. 5A, Supplementary Table T2). On the other hand, the group of patients with low expression of ADH1B was characterized by upregulation of pro-tumorigenic genes such as MYBL2, UBE2C, CENPA, TROAP, KIF18B, TPX2, HJURP, KIF2C, NEK2, and COL11A1 (Fig. 5A, Supplementary Table T2). We then performed GO enrichment analysis for the top genes upregulated in the group of samples characterized by high ADH1B expression. GO—biological process analysis revealed enrichment in the mechanisms related to cell–cell signal transduction, tissue development, and organization of extracellular structure including extracellular matrix. GO—cell composition showed enrichment mainly in collagen-containing extracellular matrix and cell membrane structure. GO—molecular function was associated mainly with binding to sulfur compounds, heparin, and glycosaminoglycans (Fig. 5B).

The next question to be analyzed was whether ADH1B expression in LUADs correlates with anti-cancer immune response. To estimate immune cell signature in TCGA samples, we have utilized the CIBERSORT algorithm. We found that cell enrichment score of plasma cells, regulatory T cells, T follicular helper cells, NK cells, and neutrophils was significantly higher in ADH1B low group. On the other hand, the score of B cells, monocytes, and mast cells was higher in ADH1B high group (Fig. 5C). These results indicate that ADH1B low samples are characterized by significantly more active inflammatory processes. Interestingly, scRNAseq data revealed that aggressive LUADs are characterized by increased proportion of T helper cells, regulatory T cells, and neutrophils, which is consistent with the data obtained using the CIBERSORT algorithm (Fig. 5D).

In addition, we estimated gene expression of different ECM proteins in TCGA samples characterized by low, intermediate, and high expression of ADH1B. We discovered that LUADs with high expression of ADH1B were characterized by decreased expression of COL1A, COL3A1, COL5A1, and COL11A1. On the other hand, the expression of ELN, LAMA2, and DCN was increased (Fig. 5E). These results support and provide additional validation of our scRNAseq findings. Altogether, it highlights the potential role of ADH1B^+^ CAFs in the determination of LUAD behavior at early stages by regulation of ECM remodeling and modulation of immune response.

## Discussion

A better understanding of the biological determinants associated with LUAD aggressiveness at the early stages of tumor development could significantly improve existing predictive models and reduce mortality^17^, while identifying actionable targets for novel therapeutics. In this work, we identified the relationship between the presence of ADH1B^+^ CAF subpopulation in the cancer tissue and LUAD aggressiveness at an early stage of tumor progression. ADH1B^+^ CAFs have decreased expression of COL1A1 and COL3A1, and increased expression of DCN, LAMA2, and ELN in comparison to other CAF subpopulations. Also, ADH1B^+^ CAFs are characterized by increased expression of IL33. Ranking the differentially expressed genes in the ADH1B^+^ subpopulation of CAFs revealed a set of top marker genes including ADH1B, CFD (DF), SELENOP (SEPP1), DCN, A2M and MFAP4. The analysis of the overall survival of LUAD patients demonstrated better survival of the patients with higher expression of these marker genes. The analysis of bulk RNAseq dataset obtained from TCGA demonstrated that high expression of the ADH1B^+^ CAF markers is associated with lower enrichment of infiltrating immune cells, and reduced expression of different types of collagens and upregulated expression of DCN, LAMA2, and ELN that is consistent with the results obtained using scRNAseq and histological assessment of LUAD samples from our cohort. The results of our research show that ADH1B^+^ CAFs correlate with LUAD behavior and can be used for the prediction of tumor aggressiveness at an early stage.

For patient stratification, we have used CANARY software as described previously^17,18^. The stratification was based on the SILA score—a computerized scoring system designed for predicting LUAD behavior and estimating patient survival. The robustness of this method has been demonstrated in several studies^16,17,32,33^. Nonetheless, a better understanding of the biological processes associated with LUAD behavior at early stages could considerably enhance existing prognostic methods. In the tumor microenvironment CAFs participate in a number of biological processes including ECM deposition and remodeling, regulation of anti-cancer immune response, angiogenesis, and tumor cell invasion^7,9–11^. Despite the active investigation of CAF function, it is still not clear if CAFs act only as a pro-tumorigenic factor or, depending on the context, if they can play an anti-tumorigenic role^12–14^. We have identified 3 distinct CAFs subpopulations in early stage LUAD tumors: FAP^+^ACTA2^+^, ADH1B^+^, and CTHRC1^+^FAP^-^. This indicates CAF diversity even at early stages of LUAD progression. Moreover, altered ratios of these CAF subtypes in the indolent and aggressive LUADs and in LUADs characterized by different histological subtypes may indicate different mechanisms of CAF activation, transition between CAF subtypes, and involvement in the determination of LUAD behavior. Grout et al.^31^ have demonstrated that enrichment of LUAD tissue with different subtypes of CAFs is determined by tumor stage and histological subtypes. Their work showed that ADH1B^+^ CAFs were enriched in early stage LUADs of the papillary subtype, whereas FAP^+^ CAFs dominated in late stage tumors of the solid histological subtype^31^. Our cohort mainly comprises samples characterized by acinar and solid histological patterns. We have identified that FAP^+^ACTA2^+^ CAFs dominate in this LUAD tissue, supporting the findings demonstrated by Grout and colleagues. Interestingly, we did not observe a significant difference in proportion between FAP^+^ACTA2^+^ and CTHRC1^+^FAP^-^ CAFs in the indolent and aggressive groups. This can be associated with the early stage of LUADs where these types of CAFs may not play a critical role. On the other hand, revealed disproportion in ADH1B^+^ CAFs could indicate their role as one of the determinants of LUAD behavior at early stages.

We have demonstrated that identified CAF subpopulations are characterized by the different activity of several signaling pathways. This may be related to either difference between distinct phenotypes or different mechanisms of CAF activation. The ADH1B^+^ subpopulation displayed low activity of signaling pathways including TGFβ, WNT, hypoxia, EGFR, TNFa, and VEGF, which implies low level of activation of these fibroblasts and places them in an intermediate state between normal fibroblasts and activated CAFs. In fact, Grout et al.^31^ have assumed that the ADH1B^+^ subpopulation of CAFs acts as a transient form between normal fibroblasts and FAP^+^aSMA^+^ CAFs. Identified similarity in gene expression between all 3 subtypes of CAFs may indicate that they could be transient forms of each other. However, this hypothesis requires further exploration.

Another important question is how exactly identified CAF subpopulations can affect the behavior of LUADs and in what biological processes are they involved in the tumor microenvironment? GO enrichment analysis showed that ADH1B^+^ CAFs participate in matrix deposition as well, but these CAFs display higher expression of DCN, LAMA2, and ELN and lower expression of different types of collagens in comparison to FAP^+^ACTA2^+^ CAFs. Interestingly, high expression of DCN, LAMA2, and ELN in lung cancer is associated with better survival^34–37^. Additionally, CAFs participate in the modulation of the anti-cancer immune response^7,15^. Beyond ECM related functions, ADH1B^+^ CAFs were characterized by increased expression of cytokines such as CXCL12, and IL33, which indicates these CAFs can be involved in immune-modulatory processes. The role of these cytokines is controversial, and it is not clear how exactly they affect the tumor microenvironment of LUADs, especially at early stages. However, several reports have shown that expression of IL33 in the tumor microenvironment correlates with better prognosis and can be associated with activation of NK cells and cytotoxic T cells^38–40^. Our results indicate that aggressive LUADs are characterized by higher immune cell enrichment including regulatory T cells and neutrophils that are associated with poor survival. In LUADs, several reports discovered CAFs that were characterized by an increased expression of different cytokines and were involved in immune response modulation processes. For example, Xing et al.^41^ identified CFD^+^CXCL14^+^ CAFs that uniquely expressed CXCL12 and CXCL14. Grout et al.^31^ identified that ADH1B^+^ CAFs had a high expression of IL34, CSF1, CCL19, CCL21, and CXCL12. Interestingly, we identified upregulation of CFD, C3 and C7 in ADH1B^+^ CAFs that are part of complement cascade. Complement has a pleotropic role in the tumor microenvironment. However, several reports indicated that complement activity can be related to the better prognosis^42,43^.

The identified subpopulation of ADH1B^+^ CAFs can be used not only for better understanding of mechanisms associated with LUAD aggressiveness but also for the development of novel predictive models. It is necessary to discover and validate a set of markers associated with ADH1B^+^ CAFs that could be identified by different methods on the transcriptomic, proteomic, and cellular levels. We have defined the top 5 marker genes (ADH1B, CFD (DF), SEPP1 (SELENOP), DCN, and A2M) that could be used for the identification of ADH1B^+^ CAFs in tumor tissues. An improved overall survival of the LUAD patients with high expression of these genes confirmed our hypothesis regarding the involvement of ADH1B^+^ CAFs in determining LUAD behavior.

Our work has several limitations. First, it is possible that the observed intratumoral cellular heterogeneity is associated with the accidental resection of healthy lung tissue due to the small size of the early-stage tumors. Second, the possible biological mechanisms that determine the presence of different CAFs subpopulations in the LUAD tissue were not investigated. Third is the small size of the patient cohort (9 indolent vs. 7 aggressive LUADs). We combined different approaches to overcome these limitations.

In conclusion, we showed that ADH1B^+^ CAFs deficiency in early-stage LUADs is associated with tumor aggressive behavior. ADH1B^+^ CAFs could potentially be used as a marker to predict LUAD behavior and for a better understanding of the biological mechanisms associated with LUAD aggressiveness. We highly suggest further investigation of this subpopulation of CAFs to better understand their origin and function in the tumor microenvironment.

### Supplementary Information

Supplementary Information.

## Data Availability

The scRNAseq dataset generated and used in this study is available on GitHub repository (https://github.com/VGeorgii/CAF_LUAD). Bulk RNA-seq data for LUAD dataset can also be downloaded from The Cancer Genome Atlas (TCGA) database (https://gdac.broadinstitute.org). Further requests for resources, information, and data should be directed to and will be provided by Michael Kammer (michael.kammer@vumc.org) or Fabien Maldonado (fabien.maldonado@vumc.org).
