# Supplementary material for: Cancer-associated fibroblasts in early-stage lung adenocarcinoma correlate with tumor aggressiveness
Source: Sci Rep. 2023 Oct 17;13:17604. doi: 10.1038/s41598-023-43296-3 (PMC10582049; doi:10.1038/s41598-023-43296-3)
Supplement: Supplementary file 1 — Supplementary Information. [file 41598_2023_43296_MOESM1_ESM.pdf]

Supplementary information

Cancer-associated fibroblasts in early-stage lung adenocarcinoma correlate with tumor aggressiveness

Vasiukov G[1], Zou Y[2], Senosain MF[2], Rahman SM Jamshedur[2], Antic SL[2], Young KM[1], Grogan EL[3], Kammer MN[2], Maldonado F[2], Reinhart-King CA[1], and Massion PP[2]

[1]Department of Biomedical Engineering, School of Engineering, Vanderbilt University, Nashville, TN.

[2]Division of Pulmonary and Critical Care Medicine, Vanderbilt University Medical Center, Nashville, TN.

[3]Division of Thoracic Surgery, Vanderbilt University Medical Center, Nashville, TN.

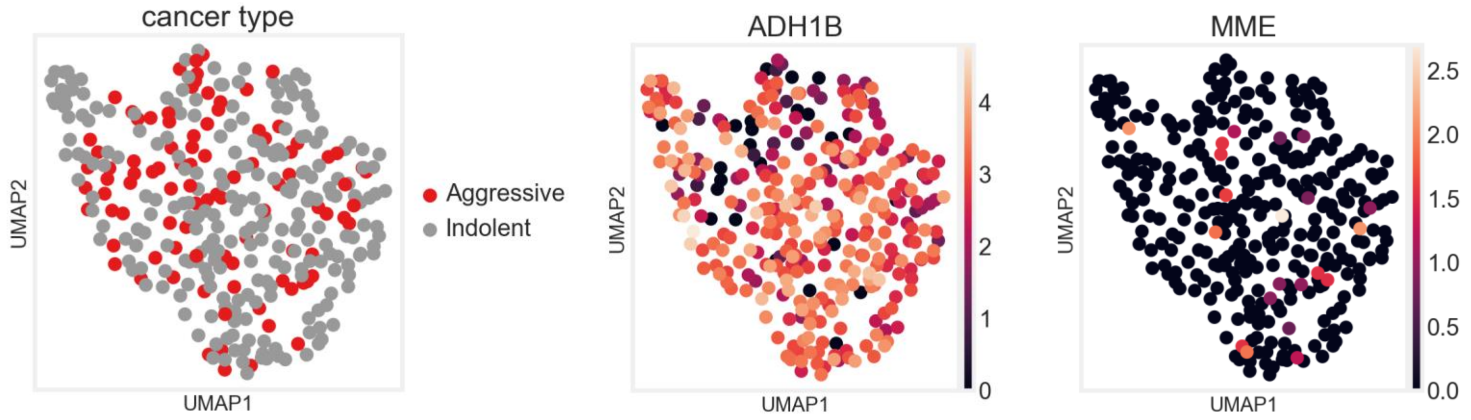

Supplementary Figure S1. UMAP representation of ADH1B⁺ CAF subcluster. Projection of samples isolated from indolent and aggressive LUADs within ADH1B⁺ CAFs subpopulation (left). Projection of ADH1B expression within ADH1B⁺ CAF subpopulation (middle). Projection of MME (CD10) expression within ADH1B⁺ CAF subpopulation (right).

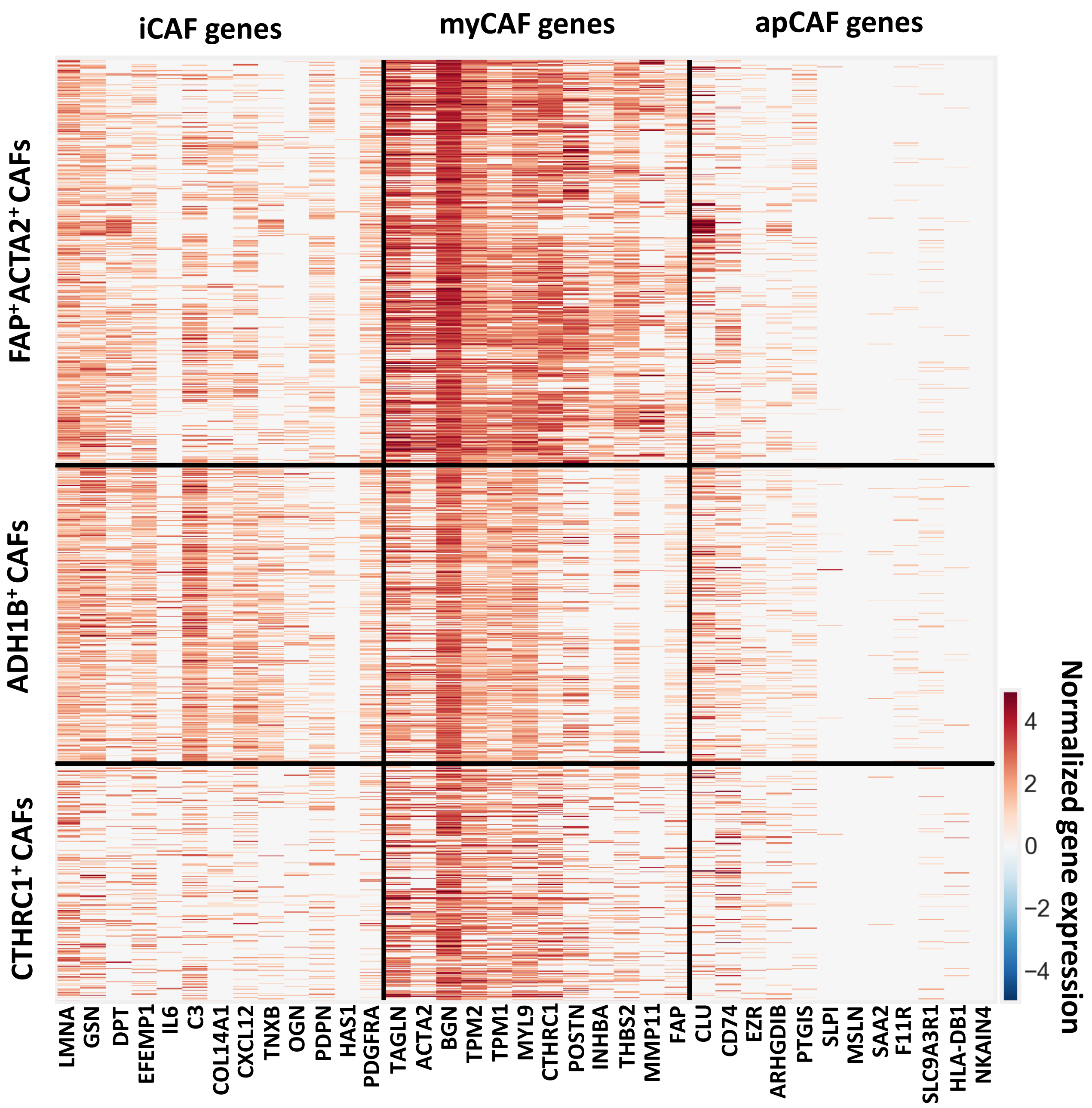

Supplementary Figure S2. Gene expression of canonical markers associated with myCAFs, iCAFs, and apCAFs in FAP$^+$ACTA2$^+$, ADH1B$^+$, and CHTRC1$^+$ CAF subpopulations. Heatmap was conducted using Python 3 (https://www.python.org).

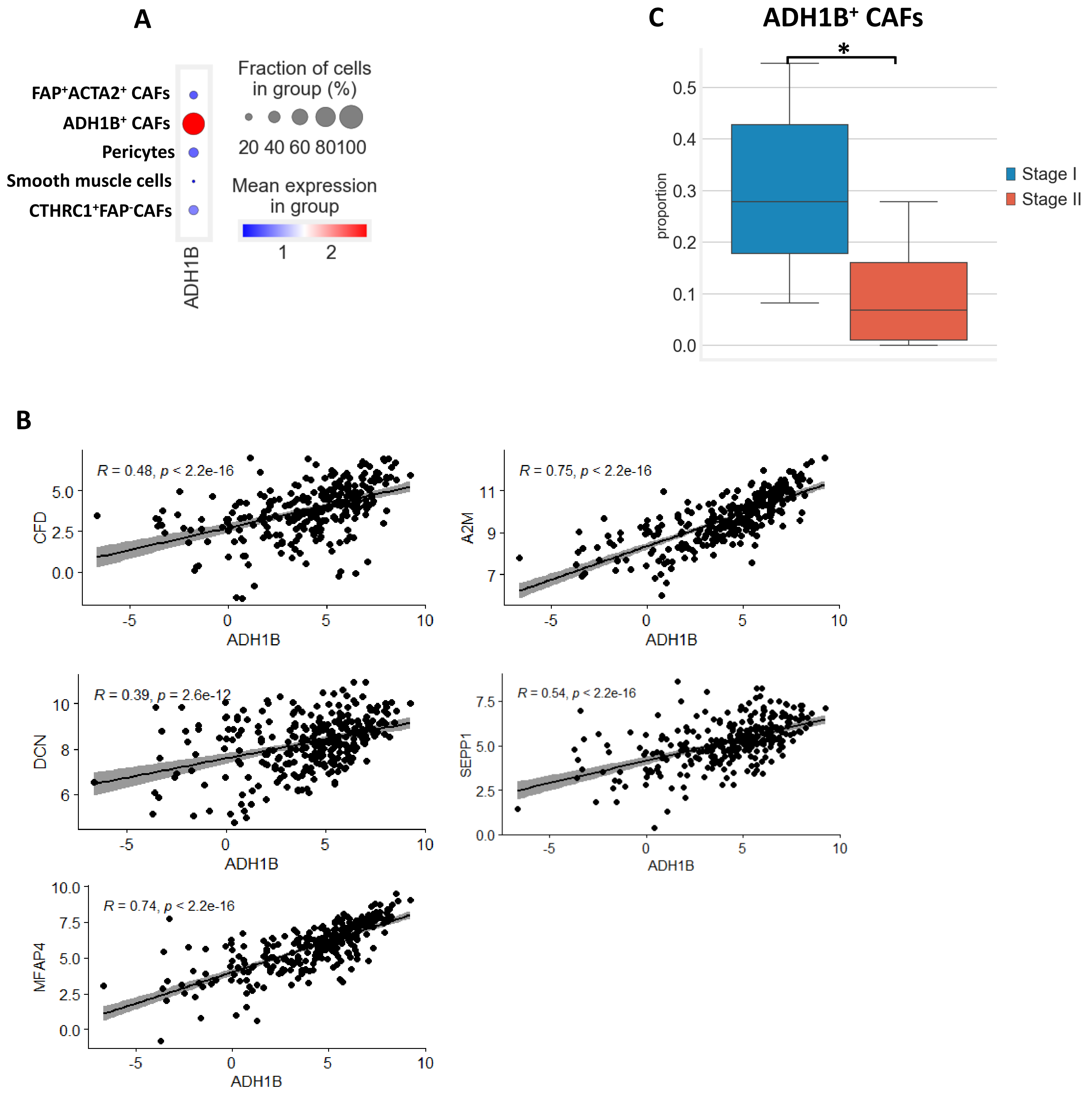

Supplementary Figure S3. A) Expression of ADH1B among stromal cells (scRNAseq data). **B)** Pearson correlation analysis between ADH1B and CFD, A2M, DCN, SEPP1, and MFAP4 (LUAD samples stage I n = 294 and stage II n = 125). **C)** Proportion of ADH1B$^+$ CAFs in the samples characterized by Stage I and Stage II (Stage I n = 7, Stage II n = 6, * - $p < 0.05$, scRNAseq data).

scRNAseq_ID	CANARY	Age	Sex	Race	Ethnicity	Smoking Status	Histological Type	8th Edition Path Stage	CT Nodule Size (mm)	CT Nodule Location	Surgery Procedure	Histological Subtype (predominant)	Histological Subtype (other)	SILA score
3	Indolent	73	Female	Caucasian	Non-Hispanic	Ex-smoker	ADC	Stage IA	NA	NA	Lobectomy	Solid	Acinar, lepidic	0.049
6	Indolent	80	Female	Caucasian	Non-Hispanic	Ex-smoker	ADC	Stage IA	23.4	LLL	Wedge Resec	Lepidic	NA	0.115
8	Indolent	62	Female	Caucasian	Non-Hispanic	Ex-smoker	ADC	Stage IA	28	RUL	Lobectomy	Acinar	Lepidic	0.23
13	Aggressive	71	Female	Caucasian	Non-Hispanic	Ex-smoker	ADC	Stage IIA	36	LUL	Lobectomy	Acinar	Solid	0.797
7	Indolent	72	Male	African American	Non-Hispanic	Ex-smoker	ADC	Stage IA	22	RUL	Wedge Resec	Solid	Acinar, lepidic	0.36
5	Aggressive	76	Female	Caucasian	Non-Hispanic	Ex-smoker	ADC	Stage IIA	46	RUL	Lobectomy	Solid	NA	0.768
1	Aggressive	86	Female	Caucasian	Non-Hispanic	Ex-smoker	ADC	Stage IB	37	LLL	Lobectomy	Acinar	Lepidic	0.622
15	Indolent	59	Male	Caucasian	Non-Hispanic	Ex-smoker	ADC	Stage IIB	12	NA	Wedge Resec	Solid	NA	0.441
11	Indolent	68	Female	Caucasian	Non-Hispanic	Current smoker	ADC	Stage IIA	NA	RUL	Lobectomy	Solid	NA	0.25
16	Aggressive	78	Female	Caucasian	Non-Hispanic	Ex-smoker	ADC	Stage IIB	NA	RUL	Lobectomy	Papillary	NA	0.853
2	Aggressive	68	Female	Caucasian	Non-Hispanic	Ex-smoker	ADC	Stage IB	27	LUL	Lobectomy	Acinar	Papillary	0.735
12	Indolent	67	Male	Caucasian	Non-Hispanic	Current smoker	ADC	Stage IB	38	RUL	Wedge Resec	Acinar	Micropapillary, Lepidic	0.493
14	Indolent	60	Female	Caucasian	Non-Hispanic	Current smoker	ADC	Stage IB	28	LUL	Lobectomy	Solid	Acinar	0.148
4	Indolent	68	Female	Caucasian	Non-Hispanic	Never smoked	ADC	Stage IB	NA	LUL	Lobectomy	Acinar	Lepidic	0.35
9	Aggressive	75	Male	Asian	Non-Hispanic	Ex-smoker	ADC	Stage IIA	33	RUL	Lobectomy	Solid	NA	0.724
10	Aggressive	83	Female	African American	Non-Hispanic	Never smoked	ADC	Stage IA3	20	LUL	Lobectomy	Mucinous acinar	NA	0.699

Supplementary table T1. Detailed patient clinical characteristics

gene_id	gene_type	gene_name	hgnc_id	havana_gene	logFC	AveExpr	P.Value	adj.P.Val
ENSG00000168484.12	protein_coding	SFTPC	HGNC:10802	OTTHUMG00000163775.2	8.2684703	5.529651	6.02E-47	5.81E-44
ENSG00000196616.14	protein_coding	ADH1B	HGNC:250	OTTHUMG00000161413.4	6.9089137	3.492732	1.52E-89	3.24E-85
ENSG00000149021.7	protein_coding	SCGB1A1	HGNC:12523	OTTHUMG00000167526.5	6.1977849	1.065363	1.19E-21	4.42E-20
ENSG00000164265.9	protein_coding	SCGB3A2	HGNC:18391	OTTHUMG00000129729.3	5.9106159	5.101728	4.52E-37	1.41E-34
ENSG00000066405.13	protein_coding	CLDN18	HGNC:2039	OTTHUMG00000159762.4	5.8191579	2.794742	1.75E-28	1.43E-26
ENSG00000161055.4	protein_coding	SCGB3A1	HGNC:18384	OTTHUMG00000130936.4	5.1997438	4.586358	2.83E-23	1.29E-21
ENSG00000168481.9	protein_coding	LGI3	HGNC:18711	OTTHUMG00000131599.5	5.1886725	-0.7596	3.93E-34	7.45E-32
ENSG00000122852.15	protein_coding	SFTPA1	HGNC:10798	OTTHUMG00000018565.6	5.1784908	8.493905	2.20E-26	1.46E-24
ENSG00000096088.16	protein_coding	PGC	HGNC:8890	OTTHUMG00000014683.7	5.1515588	4.745872	4.99E-19	1.42E-17
ENSG00000088926.14	protein_coding	F11	HGNC:3529	OTTHUMG00000150311.8	5.141428	-1.81175	1.06E-44	8.62E-42
ENSG00000165197.5	protein_coding	VEGFD	HGNC:3708	OTTHUMG00000021175.2	5.0946777	0.733812	9.05E-62	4.80E-58
ENSG00000180772.7	protein_coding	AGTR2	HGNC:338	OTTHUMG00000022243.2	5.0435063	-0.75173	9.57E-28	7.39E-26
ENSG00000156076.10	protein_coding	WIF1	HGNC:18081	OTTHUMG00000168832.5	4.9997574	1.745443	1.22E-28	1.02E-26
ENSG00000231322.5	processed_pseudogene	RPL13AP17	HGNC:36167	OTTHUMG00000155514.2	4.9865571	-2.18473	1.52E-41	8.96E-39
ENSG00000155511.18	protein_coding	GRIA1	HGNC:4571	OTTHUMG00000130148.7	4.9854795	-0.74859	2.89E-53	6.81E-50
ENSG00000185303.17	protein_coding	SFTPA2	HGNC:10799	OTTHUMG00000018566.4	4.8995557	8.925712	1.17E-25	7.04E-24
ENSG00000112936.19	protein_coding	C7	HGNC:1346	OTTHUMG00000150340.4	4.7936288	5.246794	2.26E-60	9.60E-57
ENSG00000142973.14	protein_coding	CYP4B1	HGNC:2644	OTTHUMG00000007984.9	4.7820737	4.739314	1.08E-40	5.61E-38
ENSG00000183607.10	protein_coding	GKN2	HGNC:24588	OTTHUMG00000152655.3	4.7081044	-0.13525	7.14E-24	3.44E-22
ENSG00000147655.12	protein_coding	RSPO2	HGNC:28583	OTTHUMG00000164893.4	4.6948152	-0.87762	1.11E-55	3.36E-52
ENSG00000203878.11	unprocessed_pseudogene	CHIAP2	HGNC:44463	OTTHUMG00000012173.6	4.6697603	-2.65116	2.72E-30	2.82E-28
ENSG00000171885.18	protein_coding	AQP4	HGNC:637	OTTHUMG00000131955.9	4.6268432	4.494459	2.65E-32	3.56E-30
ENSG00000161649.13	protein_coding	CD300LG	HGNC:30455	OTTHUMG00000181796.2	4.6260173	-2.41394	1.23E-50	1.86E-47
ENSG00000235584.3	lncRNA	AC008268.1	NA	OTTHUMG00000155185.6	4.5841223	-0.62933	9.39E-24	4.49E-22
ENSG00000134917.10	protein_coding	ADAMTS8	HGNC:224	OTTHUMG00000165656.3	4.5752879	1.179658	1.51E-73	1.60E-69
ENSG00000225473.1	lncRNA	ATP13A4-AS1	HGNC:41095	OTTHUMG00000156102.1	4.5699627	-1.8337	4.55E-29	4.06E-27
ENSG00000181234.9	protein_coding	TMEM132C	HGNC:25436	OTTHUMG00000163736.4	4.513181	-2.68112	1.08E-43	7.66E-41
ENSG00000125851.10	protein_coding	PCSK2	HGNC:8744	OTTHUMG00000031941.6	4.4764252	-0.88108	7.03E-16	1.39E-14
ENSG00000134020.8	protein_coding	PEBP4	HGNC:28319	OTTHUMG00000163749.6	4.4721437	2.107314	4.95E-30	4.95E-28
ENSG00000116690.13	protein_coding	PRG4	HGNC:9364	OTTHUMG00000035574.5	4.4541503	1.379751	1.20E-44	9.11E-42
ENSG00000150275.19	protein_coding	PCDH15	HGNC:14674	OTTHUMG00000018259.17	4.3677849	-2.05977	2.24E-44	1.64E-41
ENSG00000253288.2	lncRNA	AC046195.1	NA	OTTHUMG00000164173.4	4.3362258	-2.12141	1.48E-31	1.77E-29
ENSG00000124237.6	protein_coding	C20orf85	HGNC:16216	OTTHUMG00000032836.3	4.3087606	-0.21306	8.10E-18	2.05E-16
ENSG00000101938.15	protein_coding	CHRDL1	HGNC:29861	OTTHUMG00000022199.4	4.2695394	3.260159	4.12E-52	7.28E-49
ENSG00000119147.10	protein_coding	ECRG4	HGNC:24642	OTTHUMG00000130921.4	4.2471452	-0.46706	3.85E-39	1.57E-36
ENSG00000149435.12	protein_coding	GGTLC1	HGNC:16437	OTTHUMG00000032095.3	4.2411133	1.756035	4.70E-22	1.87E-20
ENSG00000179813.7	protein_coding	FAM216B	HGNC:26883	OTTHUMG00000016809.4	4.2233568	0.30624	1.93E-24	9.96E-23
ENSG00000225329.3	lncRNA	LHFPL3-AS2	HGNC:44106	OTTHUMG00000157279.2	4.1817365	0.498457	3.93E-33	6.22E-31
ENSG00000080293.10	protein_coding	SCTR	HGNC:10608	OTTHUMG00000131407.4	4.1179814	2.424665	1.06E-28	8.93E-27
ENSG00000187908.20	protein_coding	DMBT1	HGNC:2926	OTTHUMG00000019185.21	4.0930355	5.139803	1.64E-22	6.81E-21
ENSG00000170890.14	protein_coding	PLA2G1B	HGNC:9030	OTTHUMG00000169343.2	4.0614162	-0.18553	3.54E-34	6.77E-32
ENSG00000273877.4	lncRNA	AC236972.3	NA	OTTHUMG00000188638.1	4.0429348	-2.59957	2.42E-35	5.71E-33
ENSG00000133661.17	protein_coding	SFTPD	HGNC:10803	OTTHUMG00000018590.6	4.0360546	5.707513	2.29E-33	3.89E-31
ENSG00000166961.15	protein_coding	MS4A15	HGNC:28573	OTTHUMG00000167688.2	4.0346869	1.534371	4.21E-18	1.10E-16
ENSG00000162598.13	protein_coding	C1orf87	HGNC:28547	OTTHUMG00000008992.2	4.0000921	-1.89423	2.20E-22	9.07E-21
ENSG00000164161.10	protein_coding	HHIP	HGNC:14866	OTTHUMG00000161428.3	3.9854195	1.604188	7.47E-27	5.20E-25
ENSG00000170549.4	protein_coding	IRX1	HGNC:14358	OTTHUMG00000161632.2	3.97187	-1.76098	1.47E-23	6.88E-22
ENSG00000134216.19	protein_coding	CHIA	HGNC:17432	OTTHUMG00000011165.4	3.9068354	0.72851	3.46E-21	1.21E-19
ENSG00000266524.3	protein_coding	GDF10	HGNC:4215	OTTHUMG00000188319.2	3.9019445	0.572313	9.81E-45	8.33E-42
ENSG00000151892.15	protein_coding	GFRA1	HGNC:4243	OTTHUMG00000019097.4	3.8581858	1.295532	1.71E-41	9.82E-39
ENSG00000018625.15	protein_coding	ATP1A2	HGNC:800	OTTHUMG00000024080.4	3.8351452	0.270462	8.75E-55	2.32E-51
ENSG00000250978.5	lncRNA	AC079467.1	NA	OTTHUMG00000162358.1	3.8205953	-2.01327	1.60E-24	8.36E-23
ENSG00000250266.2	lncRNA	LINC01612	HGNC:51837	OTTHUMG00000160893.7	3.8198515	-2.39875	1.39E-28	1.15E-26
ENSG00000007174.18	protein_coding	DNAH9	HGNC:2953	OTTHUMG00000130383.9	3.8063378	0.03154	1.11E-19	3.35E-18
ENSG00000168079.17	protein_coding	SCARA5	HGNC:28701	OTTHUMG00000132172.4	3.780021	1.072447	1.53E-28	1.26E-26
ENSG00000241644.2	protein_coding	INMT	HGNC:6069	OTTHUMG00000167163.2	3.7701543	3.846153	1.62E-63	1.15E-59
ENSG00000171243.8	protein_coding	SOSTDC1	HGNC:21748	OTTHUMG00000090807.2	3.7650607	-0.97095	1.37E-21	5.04E-20
ENSG00000080618.17	protein_coding	CPB2	HGNC:2300	OTTHUMG00000016867.6	3.7542916	-0.29638	1.24E-20	4.09E-19
ENSG00000228723.6	lncRNA	SRGAP3-AS2	HGNC:40899	OTTHUMG00000155019.4	3.7393327	-1.72684	1.69E-16	3.62E-15
ENSG00000154080.14	protein_coding	CHST9	HGNC:19898	OTTHUMG00000179469.5	3.7258541	-0.32667	4.52E-15	8.01E-14
ENSG00000165072.10	protein_coding	MAMDC2	HGNC:23673	OTTHUMG00000019990.2	3.7111197	2.366225	3.44E-53	7.29E-50
ENSG00000162896.6	protein_coding	PIGR	HGNC:8968	OTTHUMG00000036581.2	3.7103103	7.73842	6.93E-17	1.56E-15
ENSG00000153446.16	protein_coding	C16orf89	HGNC:28687	OTTHUMG00000159314.4	3.7017723	5.983736	1.92E-24	9.91E-23
ENSG00000141052.18	protein_coding	MYOCD	HGNC:16067	OTTHUMG00000058767.6	3.6919162	0.138515	4.46E-42	2.70E-39
ENSG00000204305.14	protein_coding	AGER	HGNC:320	OTTHUMG00000031120.4	3.6904203	4.328583	9.43E-30	9.22E-28
ENSG00000120907.18	protein_coding	ADRA1A	HGNC:277	OTTHUMG00000099459.3	3.6683972	-2.85247	1.42E-37	4.93E-35
ENSG00000225383.8	lncRNA	SFTA1P	HGNC:18383	OTTHUMG00000017660.16	3.6549532	2.817776	1.56E-33	2.74E-31
ENSG00000136546.16	protein_coding	SCN7A	HGNC:10594	OTTHUMG00000154078.6	3.6516806	3.362241	9.12E-49	1.14E-45
ENSG00000261143.1	unprocessed_pseudogene	ADAMTS7P3	HGNC:49409	OTTHUMG00000172983.1	3.6472507	-1.11032	1.90E-40	9.18E-38
ENSG00000100448.4	protein_coding	CTSG	HGNC:2532	OTTHUMG00000140182.3	3.6439669	-0.65321	1.28E-38	4.67E-36
ENSG00000178473.7	protein_coding	UCN3	HGNC:17781	OTTHUMG00000017594.3	3.6411985	-1.40522	5.89E-17	1.34E-15
ENSG00000166959.8	protein_coding	MS4A8	HGNC:13380	OTTHUMG00000167686.3	3.6300085	0.394468	4.58E-15	8.11E-14
ENSG00000154198.14	unprocessed_pseudogene	CYP4Z2P	HGNC:24426	OTTHUMG00000008024.2	3.6277855	-2.14563	1.47E-26	9.98E-25
ENSG00000039537.14	protein_coding	C6	HGNC:1339	OTTHUMG00000094781.6	3.6167802	-0.17986	1.16E-21	4.34E-20
ENSG00000168878.19	protein_coding	SFTPB	HGNC:10801	OTTHUMG00000130181.7	3.6136642	11.17518	4.02E-17	9.33E-16
ENSG00000168631.13	protein_coding	MUCL3	HGNC:21666	OTTHUMG00000031104.5	3.6093202	1.440967	2.45E-16	5.10E-15
ENSG00000213088.12	protein_coding	ACKR1	HGNC:4035	OTTHUMG00000037182.8	3.6076523	2.463414	2.83E-41	1.58E-38
ENSG00000196090.12	protein_coding	PTPRT	HGNC:9682	OTTHUMG00000033040.5	3.6022525	-0.51614	2.59E-19	7.57E-18
ENSG00000248801.7	lncRNA	C8orf34-AS1	HGNC:27840	OTTHUMG00000164449.3	3.593717	1.328949	4.49E-29	4.02E-27
ENSG00000141338.14	protein_coding	ABCA8	HGNC:38	OTTHUMG00000180192.5	3.5615794	2.435155	1.71E-59	6.07E-56
ENSG00000196188.12	protein_coding	CTSE	HGNC:2530	OTTHUMG00000036121.2	3.5240008	5.923989	3.80E-17	8.86E-16
ENSG00000185008.17	protein_coding	ROBO2	HGNC:10250	OTTHUMG00000158935.9	3.5171801	2.066433	1.61E-40	7.95E-38
ENSG00000099994.11	protein_coding	SUSD2	HGNC:30667	OTTHUMG00000150792.2	3.5087971	5.965975	6.73E-31	7.37E-29
ENSG00000172987.13	protein_coding	HPSE2	HGNC:18374	OTTHUMG00000018880.3	3.4935231	-0.94441	1.96E-39	8.48E-37
ENSG00000144331.20	protein_coding	ZNF385B	HGNC:26332	OTTHUMG00000154559.11	3.4795715	2.031171	4.59E-24	2.28E-22
ENSG00000206557.6	protein_coding	TRIM71	HGNC:32669	OTTHUMG00000155778.4	3.4729884	-1.60771	1.80E-24	9.31E-23
ENSG00000030304.14	protein_coding	MUSK	HGNC:7525	OTTHUMG00000020485.7	3.4576448	-1.06763	1.68E-37	5.74E-35
ENSG00000175084.12	protein_coding	DES	HGNC:2770	OTTHUMG00000058924.4	3.453764	2.098876	1.50E-36	4.31E-34
ENSG00000131400.8	protein_coding	NAPSA	HGNC:13395	OTTHUMG00000183035.3	3.4378727	8.11785	9.83E-19	2.71E-17
ENSG00000143768.13	protein_coding	LEFTY2	HGNC:3122	OTTHUMG00000037441.2	3.4368082	-1.42643	5.92E-31	6.55E-29
ENSG00000169562.13	protein_coding	GJB1	HGNC:4283	OTTHUMG00000021797.5	3.4245633	2.149344	1.70E-16	3.62E-15
ENSG00000188817.8	protein_coding	SNTN	HGNC:33706	OTTHUMG00000158766.3	3.4200014	0.60581	2.29E-19	6.73E-18
ENSG00000111339.12	protein_coding	ART4	HGNC:726	OTTHUMG00000168738.3	3.4198119	-0.44534	1.62E-46	1.50E-43
ENSG00000118526.7	protein_coding	TCF21	HGNC:11632	OTTHUMG00000015608.2	3.414795	1.08537	5.80E-52	9.47E-49
ENSG00000225342.2	lncRNA	LRRK2-DT	HGNC:40848	OTTHUMG00000133699.2	3.4145622	1.174125	5.32E-29	4.71E-27
ENSG00000158764.7	protein_coding	ITLN2	HGNC:20599	OTTHUMG00000028605.2	3.4005357	-2.78019	5.23E-18	1.35E-16
ENSG00000225670.4	lncRNA	CADM3-AS1	HGNC:40812	OTTHUMG00000037178.4	3.3934375	-2.24678	1.77E-39	7.84E-37
ENSG00000223914.4	lncRNA	LINC02471	HGNC:53410	OTTHUMG00000059745.6	3.3858731	-2.181	1.02E-22	4.34E-21
ENSG00000161798.7	protein_coding	AQP5	HGNC:638	OTTHUMG00000169710.3	3.3846762	3.278956	2.12E-11	2.13E-10
ENSG00000122756.15	protein_coding	CNTFR	HGNC:2170	OTTHUMG00000019821.6	3.3825274	-2.21555	1.86E-32	2.58E-30
ENSG00000204610.13	protein_coding	TRIM15	HGNC:16284	OTTHUMG00000031031.4	-2.017049	-2.24153	7.11E-06	2.74E-05
ENSG00000277224.2	protein_coding	H2BC7	HGNC:4752	OTTHUMG00000014445.3	-2.0245669	-2.01089	5.15E-09	3.44E-08
ENSG00000186193.9	protein_coding	SAPCD2	HGNC:28055	OTTHUMG00000020961.4	-2.0269883	2.780878	2.69E-29	2.48E-27
ENSG00000068489.13	protein_coding	PRR11	HGNC:25619	OTTHUMG00000179362.3	-2.0273199	3.934976	4.65E-35	1.07E-32

Supplementary table T2. The differentially expressed genes in the ADH1B high vs ADH1B low group (the table continues on the next page)

gene_id	gene_type	gene_name	hgnc_id	havana_gene	logFC	AveExpr	P.Value	adj.P.Val
ENSG00000237649.8	protein_coding	KIFC1	HGNC:6389	OTTHUMG00000031209.5	-2.031008	4.163462	1.02E-30	1.10E-28
ENSG00000105173.14	protein_coding	CCNE1	HGNC:1589	OTTHUMG00000177626.4	-2.0337413	2.538491	7.40E-25	4.04E-23
ENSG00000121152.10	protein_coding	NCAPH	HGNC:1112	OTTHUMG00000130451.6	-2.0425286	3.449669	5.73E-32	7.33E-30
ENSG00000080986.13	protein_coding	NDC80	HGNC:16909	OTTHUMG00000131483.5	-2.0441228	3.090762	1.79E-31	2.13E-29
ENSG00000169607.13	protein_coding	CKAP2L	HGNC:26877	OTTHUMG00000131313.3	-2.050397	2.787396	4.57E-30	4.59E-28
ENSG00000035499.13	protein_coding	DEPDC1B	HGNC:24902	OTTHUMG00000097083.4	-2.0566217	2.387172	1.54E-27	1.17E-25
ENSG00000225077.3	lncRNA	LINC00337	HGNC:28620	OTTHUMG00000001255.5	-2.0577879	-1.39071	7.10E-21	2.41E-19
ENSG00000164362.21	protein_coding	TERT	HGNC:11730	OTTHUMG00000090357.9	-2.0591416	-1.41791	1.11E-13	1.58E-12
ENSG00000171564.12	protein_coding	FGB	HGNC:3662	OTTHUMG00000150331.3	-2.0598329	0.264574	0.005042	0.010809
ENSG00000075218.19	protein_coding	GTSE1	HGNC:13698	OTTHUMG00000150486.4	-2.0622256	2.962792	1.19E-32	1.70E-30
ENSG00000156970.13	protein_coding	BUB1B	HGNC:1149	OTTHUMG00000129877.4	-2.062591	3.136847	1.88E-34	3.68E-32
ENSG00000176383.9	protein_coding	B3GNT4	HGNC:15683	OTTHUMG00000168917.3	-2.0638941	0.311449	4.40E-17	1.02E-15
ENSG00000168243.11	protein_coding	GNG4	HGNC:4407	OTTHUMG00000040740.4	-2.0650253	0.869043	3.03E-08	1.78E-07
ENSG00000154277.13	protein_coding	UCHL1	HGNC:12513	OTTHUMG00000099377.17	-2.0659366	3.993137	2.67E-09	1.88E-08
ENSG00000129810.15	protein_coding	SGO1	HGNC:25088	OTTHUMG00000130479.7	-2.0692189	1.257049	5.54E-33	8.52E-31
ENSG00000127423.11	protein_coding	AUNIP	HGNC:28363	OTTHUMG00000007372.2	-2.0720006	1.078808	7.37E-35	1.61E-32
ENSG00000127564.17	protein_coding	PKMYT1	HGNC:29650	OTTHUMG00000128975.4	-2.0724779	2.782331	8.70E-30	8.55E-28
ENSG00000051341.14	protein_coding	POLQ	HGNC:9186	OTTHUMG00000159396.2	-2.0748944	2.235702	8.52E-28	6.70E-26
ENSG00000174343.6	protein_coding	CHRNA9	HGNC:14079	OTTHUMG00000099375.3	-2.085855	-1.999	1.39E-06	6.09E-06
ENSG00000006606.9	protein_coding	CCL26	HGNC:10625	OTTHUMG00000130403.2	-2.0930164	-1.94704	3.57E-16	7.32E-15
ENSG00000248323.7	lncRNA	LUCAT1	HGNC:48498	OTTHUMG00000162611.16	-2.1002646	2.597674	9.83E-14	1.42E-12
ENSG00000237686.7	lncRNA	AL109615.3	NA	OTTHUMG00000014752.4	-2.1011152	0.938351	2.81E-18	7.50E-17
ENSG00000131747.15	protein_coding	TOP2A	HGNC:11989	OTTHUMG00000155008.4	-2.1109311	6.404319	7.83E-32	9.83E-30
ENSG00000094804.12	protein_coding	CDC6	HGNC:1744	OTTHUMG00000133324.7	-2.1199736	3.94063	5.26E-35	1.19E-32
ENSG00000071539.14	protein_coding	TRIP13	HGNC:12307	OTTHUMG00000090349.28	-2.1224914	3.909856	2.00E-31	2.35E-29
ENSG00000112742.10	protein_coding	TTK	HGNC:12401	OTTHUMG00000015088.5	-2.1242602	2.851142	1.94E-31	2.28E-29
ENSG00000165480.16	protein_coding	SKA3	HGNC:20262	OTTHUMG00000016539.5	-2.1342524	2.409826	1.57E-36	4.45E-34
ENSG00000158402.20	protein_coding	CDC25C	HGNC:1727	OTTHUMG00000129203.6	-2.1352195	1.651071	3.04E-33	5.05E-31
ENSG00000085999.13	protein_coding	RAD54L	HGNC:9826	OTTHUMG00000007772.10	-2.1396151	1.74651	4.81E-35	1.10E-32
ENSG00000196550.10	protein_coding	FAM72A	HGNC:24044	OTTHUMG00000042552.3	-2.1431061	-0.40341	9.26E-35	1.98E-32
ENSG00000249395.4	lncRNA	CASC9	HGNC:48906	OTTHUMG00000164547.4	-2.1481146	-0.85959	3.82E-05	0.000128
ENSG00000117724.13	protein_coding	CENPF	HGNC:1857	OTTHUMG00000036955.2	-2.1481894	5.296233	7.12E-32	8.99E-30
ENSG00000138180.16	protein_coding	CEP55	HGNC:1161	OTTHUMG00000018774.2	-2.1498436	4.034367	1.79E-34	3.54E-32
ENSG00000171848.16	protein_coding	RRM2	HGNC:10452	OTTHUMG00000185555.7	-2.1540376	5.243096	6.17E-34	1.16E-31
ENSG00000006047.13	protein_coding	YBX2	HGNC:17948	OTTHUMG00000177992.3	-2.1568805	0.755712	5.44E-10	4.35E-09
ENSG00000093009.11	protein_coding	CDC45	HGNC:1739	OTTHUMG00000150386.3	-2.1591178	2.88623	8.08E-32	1.01E-29
ENSG00000275713.2	protein_coding	H2BC9	HGNC:4755	OTTHUMG00000014447.3	-2.1612602	-0.82151	3.51E-09	2.42E-08
ENSG00000100526.20	protein_coding	CDKN3	HGNC:1791	OTTHUMG00000140302.4	-2.1657528	2.60497	1.03E-32	1.50E-30
ENSG00000185306.13	protein_coding	C12orf56	HGNC:26967	OTTHUMG00000168782.5	-2.1678342	-1.36537	1.78E-07	9.23E-07
ENSG00000146670.10	protein_coding	CDCA5	HGNC:14626	OTTHUMG00000150420.3	-2.1889182	4.018007	6.09E-36	1.56E-33
ENSG00000111665.12	protein_coding	CDCA3	HGNC:14624	OTTHUMG00000169014.2	-2.1909272	2.890477	3.94E-37	1.25E-34
ENSG00000019186.10	protein_coding	CYP24A1	HGNC:2602	OTTHUMG00000032773.3	-2.1945062	3.831345	5.38E-08	3.05E-07
ENSG00000168078.10	protein_coding	PBK	HGNC:18282	OTTHUMG00000102113.6	-2.1955789	2.520354	1.76E-25	1.02E-23
ENSG00000125462.19	lncRNA	MIR9-1HG	HGNC:30780	OTTHUMG00000031022.5	-2.1990498	0.138121	2.76E-11	2.73E-10
ENSG00000083782.8	protein_coding	EPYC	HGNC:3053	OTTHUMG00000170072.3	-2.2024239	-2.07904	1.78E-08	1.09E-07
ENSG00000214145.7	lncRNA	LINC00887	HGNC:48574	OTTHUMG00000155982.8	-2.2028932	-2.32321	7.83E-16	1.54E-14
ENSG00000164651.17	protein_coding	SP8	HGNC:19196	OTTHUMG00000094788.4	-2.2116053	-2.23008	3.53E-06	1.43E-05
ENSG00000129195.16	protein_coding	PIMREG	HGNC:25483	OTTHUMG00000177832.6	-2.2313553	2.047174	6.27E-31	6.90E-29
ENSG00000224817.2	lncRNA	AC010789.1	NA	OTTHUMG00000018941.3	-2.2330194	-2.66941	6.39E-06	2.47E-05
ENSG00000066279.18	protein_coding	ASPM	HGNC:19048	OTTHUMG00000036277.2	-2.2350343	3.979319	1.62E-30	1.73E-28
ENSG00000122133.17	protein_coding	PAEP	HGNC:8573	OTTHUMG00000020914.5	-2.237637	1.590384	9.13E-05	0.000284
ENSG00000174371.17	protein_coding	EXO1	HGNC:3511	OTTHUMG00000039965.9	-2.2381901	2.720457	2.40E-33	4.05E-31
ENSG00000154839.10	protein_coding	SKA1	HGNC:28109	OTTHUMG00000132685.8	-2.23861	2.000281	4.45E-33	6.94E-31
ENSG00000126787.13	protein_coding	DLGAP5	HGNC:16864	OTTHUMG00000140310.3	-2.2386793	3.478947	4.25E-31	4.80E-29
ENSG00000188610.12	protein_coding	FAM72B	HGNC:24805	OTTHUMG00000185025.1	-2.2395783	-0.38776	3.68E-32	4.82E-30
ENSG00000118193.12	protein_coding	KIF14	HGNC:19181	OTTHUMG00000035723.2	-2.2455602	2.841456	1.14E-32	1.64E-30
ENSG00000169213.7	protein_coding	RAB3B	HGNC:9778	OTTHUMG00000008627.2	-2.2473859	1.34799	1.42E-11	1.47E-10
ENSG00000111206.13	protein_coding	FOXM1	HGNC:3818	OTTHUMG00000168118.2	-2.2511958	4.221952	4.49E-32	5.81E-30
ENSG00000109674.4	protein_coding	NEIL3	HGNC:24573	OTTHUMG00000160722.2	-2.2560574	1.318981	3.63E-23	1.64E-21
ENSG00000109805.10	protein_coding	NCAPG	HGNC:24304	OTTHUMG00000128539.4	-2.2623242	3.267883	1.95E-33	3.36E-31
ENSG00000135476.12	protein_coding	ESPL1	HGNC:16856	OTTHUMG00000169674.6	-2.2691783	3.372877	2.63E-32	3.55E-30
ENSG00000079393.20	protein_coding	DUSP13	HGNC:19681	OTTHUMG00000018516.10	-2.2709458	-1.58572	4.19E-08	2.41E-07
ENSG00000143512.13	protein_coding	HHIPL2	HGNC:25842	OTTHUMG00000037545.4	-2.2950228	0.255749	1.59E-08	9.84E-08
ENSG00000178999.13	protein_coding	AURKB	HGNC:11390	OTTHUMG00000108189.6	-2.2970476	3.1647	1.06E-32	1.54E-30
ENSG00000090889.12	protein_coding	KIF4A	HGNC:13339	OTTHUMG00000021775.2	-2.3152013	3.720927	8.82E-35	1.91E-32
ENSG00000011426.11	protein_coding	ANLN	HGNC:14082	OTTHUMG00000023143.8	-2.32048	4.923252	8.95E-34	1.65E-31
ENSG00000117650.13	protein_coding	NEK2	HGNC:7745	OTTHUMG00000037121.3	-2.3271652	3.416846	1.15E-34	2.43E-32
ENSG00000228742.11	lncRNA	LINC02577	HGNC:53749	OTTHUMG00000157590.11	-2.3284328	-0.60004	1.52E-08	9.39E-08
ENSG00000024526.17	protein_coding	DEPDC1	HGNC:22949	OTTHUMG00000009212.5	-2.3495977	2.716968	1.84E-30	1.95E-28
ENSG00000165304.8	protein_coding	MELK	HGNC:16870	OTTHUMG00000019906.8	-2.3524909	3.372253	1.47E-34	3.04E-32
ENSG00000065328.17	protein_coding	MCM10	HGNC:18043	OTTHUMG00000017694.3	-2.3599125	2.622348	7.76E-33	1.17E-30
ENSG00000143228.13	protein_coding	NUF2	HGNC:14621	OTTHUMG00000034275.4	-2.3722217	3.102986	1.52E-34	3.10E-32
ENSG00000136943.11	protein_coding	CTSV	HGNC:2538	OTTHUMG00000020314.3	-2.3834948	2.156967	5.05E-22	2.00E-20
ENSG00000142945.13	protein_coding	KIF2C	HGNC:6393	OTTHUMG00000008416.7	-2.3953897	3.934798	7.50E-39	2.95E-36
ENSG00000168779.20	protein_coding	SHOX2	HGNC:10854	OTTHUMG00000158755.7	-2.4012274	-0.24121	1.72E-17	4.18E-16
ENSG00000229544.9	protein_coding	NKX1-2	HGNC:31652	OTTHUMG00000185174.10	-2.4019223	-2.15691	2.02E-07	1.03E-06
ENSG00000089685.15	protein_coding	BIRC5	HGNC:593	OTTHUMG00000177505.9	-2.4103043	4.673208	3.65E-33	5.91E-31
ENSG00000215784.6	protein_coding	FAM72D	HGNC:33593	OTTHUMG00000074801.5	-2.4249354	-1.21069	1.90E-30	1.98E-28
ENSG00000203805.11	protein_coding	PLPP4	HGNC:23531	OTTHUMG00000019168.4	-2.4264527	0.017644	8.30E-16	1.62E-14
ENSG00000117399.14	protein_coding	CDC20	HGNC:1723	OTTHUMG00000007420.3	-2.4270961	4.519614	1.24E-34	2.60E-32
ENSG00000123485.12	protein_coding	HJURP	HGNC:25444	OTTHUMG00000059125.12	-2.4641708	3.336054	1.25E-38	4.66E-36
ENSG00000197472.15	protein_coding	ZNF695	HGNC:30954	OTTHUMG00000040707.7	-2.4765566	-1.40773	1.73E-15	3.25E-14
ENSG00000088325.16	protein_coding	TPX2	HGNC:1249	OTTHUMG00000032190.3	-2.4801105	5.223781	3.91E-37	1.25E-34
ENSG00000186185.14	protein_coding	KIF18B	HGNC:27102	OTTHUMG00000179867.2	-2.5327987	3.035	5.80E-36	1.50E-33
ENSG00000135451.13	protein_coding	TROAP	HGNC:12327	OTTHUMG00000169486.3	-2.5340774	2.943333	9.19E-36	2.27E-33
ENSG00000069482.7	protein_coding	GAL	HGNC:4114	OTTHUMG00000167890.3	-2.5358914	-1.44804	1.65E-11	1.69E-10
ENSG00000263513.5	protein_coding	FAM72C	HGNC:30602	OTTHUMG00000041029.3	-2.5925873	-1.90744	7.19E-28	5.71E-26
ENSG00000159217.10	protein_coding	IGF2BP1	HGNC:28866	OTTHUMG00000161173.3	-2.6147473	-1.18517	1.06E-07	5.74E-07
ENSG00000115163.15	protein_coding	CENPA	HGNC:1851	OTTHUMG00000097073.5	-2.65607	2.048638	7.95E-39	3.07E-36
ENSG00000185686.18	protein_coding	PRAME	HGNC:9336	OTTHUMG00000151172.4	-2.6570446	0.84424	4.38E-06	1.75E-05
ENSG00000180785.10	protein_coding	OR51E1	HGNC:15194	OTTHUMG00000157024.4	-2.6777912	-1.5625	1.13E-23	5.36E-22
ENSG00000054356.14	protein_coding	PTPRN	HGNC:9676	OTTHUMG00000133129.5	-2.6867568	-0.96883	1.94E-14	3.09E-13
ENSG00000198768.11	protein_coding	APCDD1L	HGNC:26892	OTTHUMG00000032845.5	-2.6910898	-0.52577	6.87E-16	1.36E-14
ENSG00000175063.17	protein_coding	UBE2C	HGNC:15937	OTTHUMG00000033038.3	-2.7970218	4.372043	2.54E-36	7.08E-34
ENSG00000101057.16	protein_coding	MYBL2	HGNC:7548	OTTHUMG00000033062.2	-2.8589462	4.863952	7.89E-38	2.79E-35
ENSG00000060718.22	protein_coding	COL11A1	HGNC:2186	OTTHUMG00000010872.11	-3.5976868	3.899661	6.81E-20	2.10E-18

Supplementary table T2. The differentially expressed genes in the ADH1B high vs ADH1B low group
